# Use of ichthyofauna by artisanal fishermen at two protected areas along the coast of Northeast Brazil

**DOI:** 10.1186/s13002-015-0007-5

**Published:** 2015-03-06

**Authors:** Marcia Freire Pinto, José Silva Mourão, Rômulo Romeu Nóbrega Alves

**Affiliations:** Graduate Program in Ethnobiology and Nature Conservation, Universidade Federal Rural de Pernambuco, Rua Dom Manoel de Medeiros, s/n, Dois Irmãos, 52171-900 Recife, PE Brazil; Biology Department, Universidade Estadual da Paraíba, Av. das Baraúnas, 351, Bairro Universitário, 58429-500 Campina Grande, PB Brazil

**Keywords:** Artisanal fishing, Local ecological knowledge, Conservation

## Abstract

**Background:**

Fishing is one of the oldest human activities and constitutes a source of income and livelihood for millions of people, particularly in coastal regions. This study aimed to characterize the types of fish use and test whether there is a relationship between uses of fish in the communities studied.

**Methods:**

This study was conducted during the months of January to October 2013, on the beaches of Tamandaré and Batoque, both located in Northeast Brazil. Information was collected through interviews with 75 artisanal marine fishermen on the fishes they knew and their forms of use.

**Results:**

The fishermen interviewed were male, between 22 and 84 years old, and they had been fishing for over 10 years and had a low educational level. Fishermen from Tamandaré mentioned 339 popular fish names, representing 222 taxa, while Batoque fishermen mentioned 305 popular fish names, representing 215 taxa. Six types of uses of fish were characterized: food, commercial, medicinal, handicrafts, spiritual-religious purposes and aquarium. It was found that there were multiple uses for fish and that there was a relationship between these different uses, reinforcing the importance that fish have on the culture and economic activities of fishing communities.

**Conclusions:**

Artisanal fishing should be understood as a cultural activity, because the different and multiple uses fish make up the dynamics of fishing communities. Just as in the areas of this study, some of these communities are included in protected areas and, therefore, fishermen must be involved in the development and implementation of management plans of these units.

## Background

Archaeological, historical and ethnographic studies show that aquatic resources have been exploited as sources of products useful to humans since ancient times, highlighting the importance of fishing to humankind [1,2]. Such importance has been perpetuated throughout human history, and today, millions of people worldwide depend directly or indirectly on the fishing sector as a source of income and livelihood [3]. In Brazil alone, there are over a million fishermen located in the vicinity of marine and freshwater environments, from north to south [4].

However, like any other form of exploitation of natural resources, fishing causes pressure on the species caught, underscoring the urgent need to search for strategies for sustainable use of resources to enable the continuity of artisanal fisheries, the production of which in recent years has suffered a drastic decline [5]. This has caused a global crisis in the fisheries sector, strongly affecting the quality of life and sustainability of social and economic activities of people of the sea, mainly artisanal fishermen [6].

The uncontrolled exploitation of natural resources required conservation measures, which were proposed in 1992, in the Convention on Biological Diversity (CBD) [7]. One of the actions for in situ conservation was proposed by the CBD to establish a system of protected areas or areas where special measures would be taken to conserve biological diversity [7]. Accordingly, the Brazilian government, by Law No. 9985/2000 establishing the National System of Conservation Units of Nature [8], initiated a process for the creation of conservation units in the country.

However, the implementation of these protected areas has caused environmental conflicts, especially in those areas where there is overlap with the territory of traditional communities. To minimize these conflicts, after the Conference of the Parties to the Convention on Biological Diversity in 2004, the Brazilian government created the National Plan for Protected Areas [9], which establishes guidelines for environmental conservation based on the involvement of the people in and around the Conservation Units.

One relevant aspect in the definition of traditional cultures, among them the culture of artisanal fishermen, is the existence of systems for the management of natural resources, marked by respect for natural cycles and their exploitation within the recovery capacity of species used [10]. In this sense, the integration of these cultures with the environment can be an efficient way of preserving the ecological system, since their interests rest on the maintenance of ecosystems from which they derive their daily livelihood [11].

Given the scenario described above, the analysis of interactions between humans and fish through ethnoichthyological studies, is essential to think about ways of sustainable use, allowing the preservation of ichthyofaunal resources and the maintenance of the fishing culture, especially in protected areas. Ethnoichthyology aims to describe the knowledge about fish of a particular social group [12], providing support for the conservation of fish populations, by recording, recognizing and appreciating the ecological knowledge of fishermen [13].

The usefulness of fisheries resources for humans is diverse, especially as a protein source. Nevertheless, fish are used for various purposes, including commercial, handicrafts and medicinal purposes [13-15]. Most ethnoichthyological studies in Brazil have focused on fish used for used for food [16-19], and there are few studies on other uses of fish.

The present study was conducted in two different fishing communities on the northeastern coast of Brazil, with the following aims: i) to document and compare the richness of fish species according to the ichthyological knowledge of fishermen in the areas surveyed; ii) to characterize the types of fish use; iii) to assess the conservation status of the species recorded; and iv) to test whether there is a relationship between uses of fish in the communities studied. It was expected that the main use of fish was for food, and that other uses (medicinal purposes, making crafts, magical-religious purposes and aquarium) were associated with the byproducts of those fish used for food.

## Methods

### Study areas

**Figure 1:**
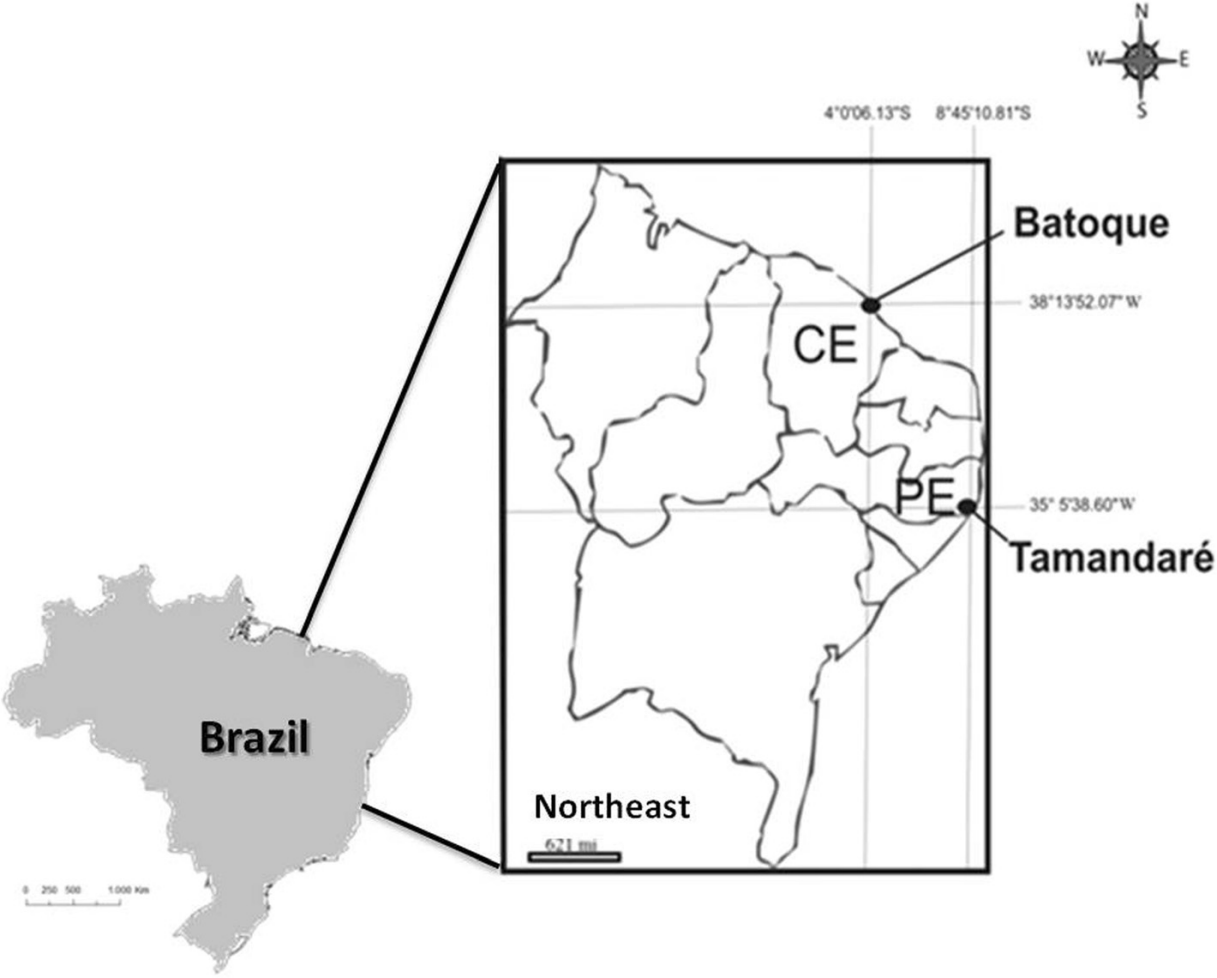
**Location beaches Tamandaré (PE) and Batoque (CE), on the coast of Northeast Brazil.**

The research was conducted with artisanal fishermen of Tamandaré Beach, in Pernambuco State, and Batoque Beach, in Ceará State, both on the coast of Northeast Brazil (Figure 1). Tamandaré Beach (8°45'10.81"S and 35°5'38.60"W) is located in the municipality of Tamandaré on the southern coast of Pernambuco, 110 km from the capital, Recife. The municipality of Tamandaré has 20,715 inhabitants [20] and is one of the major tourist centers of the Northeast, with infrastructure to meet the needs of natives, tourists and researchers. It is also harbors the Center for Research and Management of Fisheries Resources of the Northeast Coast (CEPENE), the Institute of the Environment and Renewable Natural Resources (IBAMA) and the Coastal Reef Institute (linked to the Federal University of Pernambuco), which influence the development and oversight of local artisanal fisheries.

Tamandaré Beach is in two protected areas: Municipal Natural Park of Fort Tamandaré and Coral Coast Environmental Protected Area. The Coral Coast Environmental Protection Area is the first and largest federal conservation unit to protect part of the coastal reefs.

Batoque Beach (4°0’06.13”S and 38°13’52.07”W) is located in the municipality of Aquiraz, east coast of Ceará State, and is 54 km from the capital, Fortaleza. Aquiraz has a population of 72,628 inhabitants [20], and it is characterized by high real estate speculation and tourism on its beaches. However, Batoque is a small fishing village, part of the Extractive Reserve (RESEX) of Batoque, created by Presidential Decree of June 5, 2003. RESEX covers 601 hectares and has a population of approximately 460 inhabitants and a few commercial establishments.

The study areas were chosen because they are in protected areas and have artisanal fishing as one of the main economic activities. Furthermore, the two beaches show socioeconomic and environmental disparities, suggesting the existence of differences in fishing activity and ichthyological knowledge of the fishermen. According to information from the Z-5 colony of fishermen, there are 40 registered artisanal fishermen fishing at Tamandaré Beach in motor boats or rowboats. In Batoque, according to the Association of Fishermen and shellfish gatherers of the Batoque RESEX, there are 48 artisanal fishermen who fish primarily in sail boats, locally called “jangadas”. The study was conducted with 36 fishermen (90%) of Tamandaré Beach and 39 (81%) of Batoque Beach, totaling 75 artisanal marine fishermen, whose catch is mostly fish.

### Data collection

The data related to socioeconomic information and knowledge about the ichthyofauna richness recognized and used locally were obtained using structured and semi-structured interviews, complemented by free interviews [21] and informal conversations with the fishermen. Before each interview, we explained the objectives and nature of the study and requested permission for the interviews. The study was approved by the Ethics Committee on Research Involving Human Subjects at the Federal University of Pernambuco (CAAE 05757512.5.0000.5208).

The first contacts with fishermen of Tamandaré and Batoque were through the aid of key informants [22] selected among all informants to cooperate more actively in research and to facilitate the contact with the community. Other respondents were indicated by the “snowball” technique [23], in a stratified sample that included only marine fishermen of each study location.

The interviews took place monthly from January to October 2013 and were conducted in the homes of the fishermen or on the beach and had an average duration of 40 minutes per respondent. To understand the socioeconomic context of the fishing activity, we initially determined the profile of the fishermen on the basis of structured forms with reference to the name, age, schooling and income of fishermen, economic activities developed by them, and also the time they practiced their fishing activity.

Fishermen were asked about the fish they knew and used or were aware of any type of use for the fish, through three supplementary interviewing techniques - Free listed items [24], Nonspecific prompting and Reading Back the list [25]. Direct observations and informal interviews with fishermen were conducted during the fish landing.

The identification of fish was performed using specimens as well as photographs and drawings shown to the fishermen, as proposed by Lopes, Silvano and Begossi [26]. The specimens were identified with the aid of information from the database of the Fisheries Statistics Project (ESTATPESCA) of FishBase (www.fishbase.org) and Coastal Reefs Institute, as well as research on fish populations in Northeast Brazil [27,28]. Cross identification was carried out, where fishermen identified specimens of fish previously identified by other respondents [29]. This technique was applied with three fishermen in each community, which demonstrated greater knowledge, from the number of citations and descriptions of fish in interviews.

### Data analysis

Analyses of species richness were performed using Primer 6.1 software. The chi-square test (α = 5%), using BioEstat 5.3 software, was performed to determine any differences between the two fishing communities in richness of the fish fauna. The use value (UV) was determined for each species recorded [30], which allowed us to demonstrate the relative importance of the species known locally, regardless of the opinion of the researcher. UV was calculated using the following formula: UV = ΣU/n, where U = number of citations per species and n = number of informants.

Additionally, we checked the conservation status of the species recorded in accordance with the list of endangered species of the International Union for Conservation of Nature (IUCN) in 2014 [31], the 2008 red list of the Brazilian Institute of Environment and Renewable Natural Resources [32], and the 2004 national list of species of aquatic invertebrates and fish overexploited or threatened overexploitation [33].

In cross-identification, the number of matches between the identifications of fishermen was considered. The fish that showed disagreements in identification were later identified by the consensus of a group of three to five fishermen.

In order to verify the similarity of the types of uses of fish, using Primer 6.1 software, it was performed cluster analysis with Euclidean Distance, represented by horizontal dendograms. The vertical lines represent the groups attached in descending order of similarity, while the horizontal lines indicate the distances between groups that were formed. The lower the value of the Euclidean Distance, the greater is the similarity between clusters.

## Results

### Socioeconomic profile of fishermen

The fishermen interviewed (n = 75) were male and aged between 22 and 84 years, with an average of 55 and 50 years in Tamandaré at Batoque, respectively. Some factors, according to the fishermen, explained why the disinterest of the younger men with fishing and the search for new employment opportunities, such as: 1) lack of government investment and subsidies for the storage, processing and selling of local fish; 2) the low market value of local fish, and 3) the weak supervision of illegal and commercial fishing.

Only six fishermen were less than 30 years old and fishing in the Batoque Beach, where there are few employment opportunities in comparison with the Tamandaré Beach. The average monthly income of fishermen is R$ 326 for Tamandaré and R$ 530 for Batoque. According to the fishermen, income depends on the amount and quality of fish, as well as weather and sea, which influence fishing. Of the total respondents, 88% work exclusively in fishing, and 12% work in other activities such as masons, carpenters, sailors, merchants or home custodians (people who are in charge of taking care of a house belonging to people who do not live in the community). Among the interviewed fishermen, 11 fished less than 20 years and 64 fished for over 20 years.

With regard to education, 15 Tamandaré fishermen were illiterate and 21 did not complete elementary school. In Batoque, 17 fishermen were illiterate, 20 did not complete primary school, and two did not complete high school.

### Fishermen’s knowledge of the fish community richness

Fishermen Tamandaré mentioned 339 popular names of fish, representing 222 taxa (202 species and 20 identified at the genus level). Batoque fishermen mentioned 305 popular names of fish, representing 215 taxa (194 species and 21 identified at the genus level). There was no statistically significant difference (p = 0.737) between the two communities with regard to fish community richness. In short, the fish that had 100% confirmation by fishermen in the cross-identification technique were recorded at the species level, whereas fish that had divergent identifications were recorded at the genus level.

Additionally, it was not possible to identify 24 fish cited by the Tamandaré fishermen and 18 fish cited by the Batoque fishermen, due to the difficulty they had in identifying fish through photographs and drawings, and also because it was not possible to collect them. There were cases where a popular name of a fish corresponded to one species and where a single species corresponded to several common names.

### Types of uses of fish

Fishermen mentioned six types of uses of fish: food, commercial, medicinal, handicrafts, spiritual-religious purposes and aquarium. It is noteworthy that the fishermen cited commercial use only directed at fish for food consumption.

There were 207 species with use citations in Tamandaré and 209 in Batoque (Tables 1 and 2). The general use value (considering all the citations for different uses) of these species ranged from 0.02 to 1.94 in Tamandaré, and 0.02 to 1.92 in Batoque.

**Table 1:** **Fish species recorded through interviews with marine artisanal fishermen of Tamandaré Beach, Pernambuco, Brazil**

**Family**	**Scientific name**	Name in English*	**Local name**	**IUCN (2014)**	**IBAMA (2004)**	**F**	**Co**	**Med**	**H**	**S-R**	**Aq**	**Use values**
Acanthuridae	*Acanthurus bahianus* (Castelnau, 1855)	Ocean surgeon	Caraúna	LC		x	x					0,61
Acanthuridae	*Acanthurus chirurgus* (Bloch, 1787)	Doctorfish	Caraúna-preta	LC		x	x					0,06
Acanthuridae	*Acanthurus coeruleus* (Bloch & Schneider, 1801)	Blue tang surgeonfish	Caraúna-azul	LC		x	x					0,06
Achiridae	*Achirus lineatus* (Linnaeus, 1758)	Lined sole	Sóia-redonda	NE								0,00
Albulidae	*Albula nemoptera* (Fowler, 1911)	Threadfin bonefish	Ubarana-boca-de-rato	DD		x	x					0,11
Albulidae	*Albula vulpes* (Linnaeus, 1758)	Bonefish	Ubarana	NT		x	x					0,06
Antennariidae	*Antennarius multiocellatus* (Valenciennes, 1837)	Longlure frogfish	Aniquim-mole	NE								0,00
Ariidae	*Genidens genidens* (Cuvier, 1829)	Guri sea catfish	Bagre-ariaçu; Bagre-giriaçu; giruaçu; juruaçu; Bagre-branco; Bagre-miguel-raio	LC		x	x					0,94
Ariidae	*Bagre bagre* (Linnaeus, 1766)	Coco sea catfish	Bagre-bardecha; Bagre-bandeira; Bagre-fita	NE		x	x					0,39
Ariidae	*Aspistor quadriscutis* (Valenciennes, 1840)	Bressou sea catfish	Bagre-amarelo; Bagre-mestre-mané	NE		x						0,33
Ariidae	*Sciades proops* (Valenciennes, 1840)	Crucifix sea catfish	Bagre-corre-costa	NE		x	x					0,17
Ariidae	*Cathorops spixii* (Agassiz, 1829)	Madamango sea catfish	Bagre-bandim; Bagre-manguim	NE		x	x					0,11
Ariidae	*Sciades herzbergii* (Bloch, 1794)	Pemecou sea catfish	Bagre-barba-roxa	NE		x						0,06
Balistidae	*Balistes vetula* (Linnaeus, 1758)	Queen triggerfish	Cangulo-amarelo; Cangulo-verdadeiro; cangulo-do-papo-amarelo; Cangulo-papo-louro; Cangulo-azul	VU		x	x					0,89
Balistidae	*Balistes capriscus* (Gmelin, 1788)	Grey triggerfish	Cangulo-fernando; Cangulo-fernandi; cangulo-branco; Cangulo-papo-branco; Cangulo-patriota	NE	x	x	x					0,72
Balistidae	*Canthidermis sufflamen* (Mitchill, 1815)	Ocean triggerfish	Cangulo-mané-do-arroio; Cangulo-mané-de-arroz; Cangulo-preto; Cangulo-guiné	NE		x	x					0,50
Balistidae	*Melichthys niger* (Bloch, 1786)	Black triggerfish	Cangulo-mané-do-arroio; Cangulo-mané-de-arroz; Cangulo-preto; Cangulo-guiné	NE		x	x					0,50
Batrachoididae	*Amphichthys cryptocentrus* (Valenciennes, 1837)	Bocon toadfish	Pacamon; Pocomão	LC		x	x					0,28
Batrachoididae	*Batrachoides surinamensis* (Bloch & Schneider, 1801)	Pacuma toadfish	Pacamon; Pocomão	NE		x	x					0,28
Batrachoididae	*Thalassophryne nattereri* (Steindachner, 1876)	Trinidad Tob	Pacamon; Pocomão	NE		x	x					0,28
Belonidae	*Tylosurus acus* (Lacepède, 1803)	Agujon needlefish	Agulhão-branco	NE		x	x					0,06
Belonidae	*Strongylura timucu* (Walbaum, 1792)	Timucu	Agulhão-espinha-verde	NE		x	x					0,17
Bothidae	*Bothus* spp.	Plate fish	Sóia	NE								0,00
Carangidae	*Elagatis bipinnulata* (Quoy & Gaimard, 1825)	Rainbow runner	Arabaiana; Gurubatã; Guiubatá; Peixe-rei	NE		x	x					1,94
Carangidae	*Caranx crysos* (Mitchill, 1815)	Blue runner	Guarassuma; garassuma; Chincharro; Xerelete	LC		x	x					1,33
Carangidae	*Decapterus macarellus* (Cuvier, 1833)	Mackerel scad	Garapau	NE		x	x					1,11
Carangidae	*Caranx latus* (Agassiz, 1831)	Horse-eye jack	Garacimbora; Aracimbora; Garachimbora; Guachimbora	NE		x	x					0,67
Carangidae	*Alectis ciliaris* (Bloch, 1787)	African pompano	Galo-de-penacho; Galo-do-alto; Galo-de-fita	LC		x	x					0,33
Carangidae	*Caranx bartholomaei* (Cuvier, 1833)	Yellow jack	Xaréu-amarelo	NE		x	x					0,33
Carangidae	*Caranx hippos* (Linnaeus, 1766)	Crevalle jack	Xaréu-branco	NE		x	x					0,33
Carangidae	*Selene vomer* (Linnaeus, 1758)	Lookdown	Galo-de-penacho; Galo-do-alto; Galo-de-fita	NE		x	x					0,33
Carangidae	*Caranx ruber* (Bloch, 1793)	Bar jack	Xaréu preto; Garajuba-branca	NE		x	x					0,28
Carangidae	*Trachinotus* spp.	Floripa pompano	Pampo; Piraroba	NE		x	x					0,22
Carangidae	*Caranx* sp.		Capitão-garajuba	Sem avaliação		x	x					0,17
Carangidae	*Chloroscombrus chrysurus* (Linnaeus, 1766)	Atlantic bumper	Pelombeta; Pilombeta; Palombeta	NE		x	x					0,17
Carangidae	*Seriola dumerili* (Risso, 1810)	Greater amberjack	Olhete; Arabaiana-cachorro	NE		x	x					0,17
Carangidae	*Seriola rivoliana* (Valenciennes, 1833)	Longfin yellowtail	Arabaiana-chata	NE		x	x					0,17
Carangidae	*Oligoplites palometa* (Cuvier, 1832)	Maracaibo leatherjacket	Tibiro; Timbiro	NE		x	x					0,11
Carangidae	*Oligoplites saliens* (Bloch, 1793)	Castin leatherjacket	Tibiro; Timbiro	NE		x	x					0,11
Carangidae	*Oligoplites saurus* (Bloch & Schneider, 1801)	Leatherjacket	Tibiro; Timbiro	NE		x	x					0,11
Carangidae	*Seriola lalandi* (Valenciennes, 1833)	Yellowtail amberjack	Arabaiana-amarela; Arabaiana-preta	NE		x	x					0,11
Carangidae	*Seriola fasciata* (Bloch, 1793)	Lesser amberjack	Arabaiana-roliça; Arabaiana-branca	NE		x	x					0,06
Carcharhinidae	*Galeocerdo cuvier* (Péron & Lesueur, 1822)	Tiger shark	Cação-pintadinho; Cação-pintado; Jaguara; Cação-tigre; Tubarão-tigre	NT		x	x					0,72
Carcharhinidae	*Carcharhinus falciformis* (Müller & Henle, 1839)	Silky shark	Cação-aba-preta; Cação-sicurí; Galha-preta; Tubarão-galha-preta; Tubarão-aba-preta; Cação-flamengo	NT		x	x					0,61
Carcharhinidae	*Carcharhinus limbatus* (Müller & Henle, 1839)	Blacktip shark	Cação-aba-preta; Cação-sicurí; Galha-preta; Tubarão-galha-preta; Tubarão-aba-preta; Cação-flamengo	NT		x	x					0,61
Carcharhinidae	*Carcharhinus leucas* (Müller & Henle, 1839)	Bull shark	Cação-cabeça-chata; Tubarão-cabeça-chata	NT		x	x					0,44
Carcharhinidae	*Prionace glauca* (Linnaeus, 1758)	Blue shark	Cação-azul; Cação-barriga-mole	NT	x	x	x					0,33
Carcharhinidae	*Carcharhinus* spp.		Cação-lombo-preto	Sem avaliação		x	x					0,06
Carcharhinidae	*Carcharhinus* sp.		Cação-toalha	Sem avaliação								0,00
Carcharhinidae	*Rhizoprionodon lalandi * (Valenciennes, 1839)	Brazilian sharpnose shark	Cação-verga-de-ouro	DD								0,00
Carcharhinidae	*Rhizoprionodon porosus* (Richardson, 1836)	Caribeean sharpnose Shark	Cação-verga-de-ouro	LC								0,00
Centropomidae	*Centropomus pectinatus* (Poey, 1860)	Tarpon snook	Camurim-branco; Camurim-impim; Camurim-tábua	NE		x	x					0,67
Centropomidae	*Centropomus undecimalis* (Bloch, 1792)	Common snook	Camurim-açu; Camurim-corcundo; Camurim-preto	NE		x	x					0,61
Chaetodontidae	*Chaetodon* spp.	Spotfin butterflyfish	Parum-jandáia; Peixe-prato; Pintado	LC								0,00
Clupeidae	*Opisthonema oglinum* (Lesueur, 1818)	Atlantic thread herring	Sardinha; Sardinha-azul; Sardinha-de-gaia	NE		x	x					0,56
Clupeidae	*Harengula jaguana* (Poey, 1865)	Scaled herring	Sardinha-cascuda; Sardinha-casca-grossa	NE		x	x					0,44
Clupeidae	*Sardinella aurita* (Valenciennes, 1847)	Round sardinella	Sardinha-maromba	NE		x	x					0,11
Clupeidae	*Sardinella brasiliensis* (Steindachner, 1879)	Brazilian sardinella	Sardinha-roliça	NE		x	x					0,06
Coryphaenidae	*Coryphaena equiselis* (Linnaeus, 1758)	Pompano dolphinfish	Dourado; Dourado-azedinho	LC		x	x					1,86
Coryphaenidae	*Coryphaena hippurus* (Linnaeus, 1758)	Common dolphinfish	Dourado; Dourado-cabeça-de-bolina	LC		x	x					1,86
Cynoglossidae	*Symphurus* spp.	Spottedfin tonguefish	Sóia-linguado; Linguado	NE								0,00
Dasyatidae	*Dasyatis guttata* (Bloch & Schneider, 1801)	Longnose stingray	Arraia-branca; Arraia-couro-de-lixa	DD		x	x					0,33
Dasyatidae	*Dasyatis americana* (Hildebrand & Schroeder, 1928)	Southern stingray	Arraia-mijona	DD		x						0,28
Dasyatidae	*Dasyatis* sp.		Arraia-de-pedra; Arraia-de-croa	LC		x	x					0,22
Diodontidae	*Chilomycterus antillarum* (Jordan & Rutter, 1897)	Web burrfish	Baiacu-espinho	NE				x				0,22
Diodontidae	*Chilomycterus spinosus spinosus* (Linnaeus, 1758)		Baiacu-espinho	NE				x				0,22
Echeneidae	*Echeneis naucrates* (Linnaeus, 1758)	Live sharksucker	Piolho	NE		x						0,17
Echeneidae	*Remora remora* (Linnaeus, 1758)	Shark sucker	Piolho	NE		x						0,17
Echinorhinidae	*Echinorhinus brucus* (Bonnaterre, 1788)	Bramble shark	Peixe-prego	DD		x	x					0,17
Elopidae	*Elops saurus* (Linnaeus, 1766)	Ladyfish	Ubarana-boca-larga	LC		x	x					0,11
Engraulidae	*Anchoa januaria* (Steindachner, 1879)	Rio anchovy	Manjuba	NE		x	x					0,06
Engraulidae	*Anchoa tricolor* (Spix & Agassiz, 1829)	Piquitinga anchovy	Manjuba	NE		x	x					0,06
Engraulidae	*Lycengraulis grossidens* (Spix & Agassiz, 1829)	Atlantic sabretooth anchovy	Arenque-amarelo	NE		x	x					0,06
Engraulidae	*Lycengraulis batesii* (Günther, 1868)	Bates' sabretooth anchovy	Arenque-boca-larga; Arenque-boca-de-velho	NE		x	x					0,06
Ephippidae	*Chaetodipterus faber* (Broussonet, 1782)	Atlantic spadefish	Enxada; Parum-branco	NE		x	x					0,17
Exocoetidae	*Cypselurus cyanopterus* (Valenciennes, 1846 )	Margined flyingfish	Avuador-holandês	NE		x	x					0,22
Exocoetidae	*Hirundichthys affinis* (Günther, 1866)	Fourwing flyingfish	Avuador-da-pesca; Peixe-avuador-pequeno	NE		x	x					0,11
Exocoetidae	*Exocoetus volitans* (Linnaeus, 1758)	Tropical two-wing flyingfish	Avuador-do-alto; Peixe-avuador-grande	NE		x	x					0,06
Fistulariidae	*Fistularia petimba* (Lacepède, 1803)	Red cornetfish	Agulhão-trombeta	NE		x						0,03
Gempylidae	*Gempylus serpens* (Cuvier, 1829)	Snake mackerel	Espada-preta	NE		x	x					0,06
Gerreidae	*Diapterus rhombeus* (Cuvier, 1829)	Caitipa mojarra	Carapeba	NE		x	x					0,72
Gerreidae	*Eugerres brasilianus* (Cuvier, 1830)	Brazilian mojarra	Carapeba	NE		x	x					0,72
Gerreidae	*Diapterus auratus* (Ranzani, 1842)	Irish mojarra	Carapitinga; Carapeba	NE		x	x					0,67
Gerreidae	*Eucinostomus sp.*	Slender mojarra	Carapicu	NE		x	x					0,17
Gerreidae	*Gerres cinereus* (Walbaum, 1792)	Yellon fin mojarra	Carapicu	NE		x	x					0,17
Gerreidae	*Eucinostomus havana* (Nichols, 1912)	Bigeye mojarra	Carapicu-roliço	NE		x	x					0,06
Gerreidae	*Eucinostomus gula* (Quoy & Gaimard, 1824)	Jenny mojarra	Carapicu-açu	NE		x	x					0,06
Ginglymostomatidae	*Ginglymostoma cirratum* (Bonnaterre, 1788)	Nurse shark	Cação-lixa	DD		x	x					0,50
Gymnuridae	*Gymnura micrura* (Bloch & Schneider, 1801)	Smooth butterfly ray	Arraia-manteiga	DD		x	x					0,39
Haemulidae	*Haemulon plumierii* (Lacepède, 1801)	White grunt	Biquara	NE		x	x					1,17
Haemulidae	*Anisotremus surinamensis* (Bloch, 1791)	Black margate	Salema-açu; Salema-preta; Salema-pintada; Avô-de-pirambu; Pirambu	NE		x	x					0,56
Haemulidae	*Haemulon parra* (Desmarest, 1823)	Sailor's grunt	Cancanhé	NE		x	x					0,50
Haemulidae	*Anisotremus virginicus* (Linnaeus, 1758)	Porkfish	Frade; Salema-feiticeira; Salema-freada; Salema-amarela	NE		x	x					0,33
Haemulidae	*Orthopristis ruber* (Cuvier, 1830)	Corocoro grunt	Cabeça-de-coco; cabeça-dura; Canguito	NE		x	x					0,33
Haemulidae	*Pomadasys corvinaeformis* (Steindachner, 1868)	Roughneck grunt	Coró-branco; Coróqui-branco	NE		x	x					0,19
Haemulidae	*Conodon nobilis* (Linnaeus, 1758)	Barred grunt	Coró-amarelo; Coró-rajado; Coróqui-amarelo	NE		x	x					0,14
Haemulidae	*Haemulon aurolineatum* (Cuvier, 1830)	Tomtate grunt	Xira-roliça	NE		x	x					0,11
Haemulidae	*Haemulon album* (Cuvier, 1830)	White margate	Xira-branca	NE		x						0,11
Haemulidae	*Haemulon chrysargyreum* (Günther, 1859)	Smallmouth grunt	Sapuruna	NE		x	x					0,11
Haemulidae	*Haemulon squamipinna* (Rocha & Rosa, 1999)		Xira listradim; xira-amarela	NE		x						0,11
Haemulidae	*Haemulon steindachneri* (Jordan e Gilbert, 1882)	Chere-chere grunt	Macasso; Omacasso	LC		x	x					0,06
Haemulidae	*Haemulon macrostomum* (Günther, 1859)	Spanish grunt	Cavalo-pedrez; Xirão	NE		x	x					0,06
Hemiramphidae	*Hemiramphus balao* (Lesueur, 1821)	Balao halfbeak	Agulha-preta	NE		x	x					0,83
Hemiramphidae	*Hyporhamphus roberti* (Valenciennes, 1847)	Slender halfbeak	Agulha-branca	LC		x	x					0,72
Hemiramphidae	*Hemiramphus brasiliensis* (Linnaeus 1758)	Ballyhoo halfbeak	Agulha-rabo-de-fogo	NE		x	x					0,28
Holocentridae	*Holocentrus adscensionis* (Osbeck, 1765)	Squilrrelfish	Mariquita; jaguriçá; Mariquita-verdadeira	NE		x	x					0,56
Holocentridae	*Myripristis jacobus* (Cuvier, 1829)	Blackbar soldierfish	Vovozinha	NE		x	x					0,06
Istiophoridae	*Kajikia albida* (Poey, 1860)	Atlantic White marlin	Agulhão-roliço; Atum; Agulhão-negro	VU		x	x					0,61
Istiophoridae	*Makaira nigricans* (Lacepède, 1802)	Blue marlin	Agulhão-roliço; Atum; Agulhão-negro	VU		x	x					0,61
Istiophoridae	*Tetrapturus pfluegeri* (Robins & de Sylva, 1963)	Longbill spearfish	Agulhão-marli	LC		x	x					0,17
Istiophoridae	*Istiophorus albicans* (Latreille, 1804)	Atlantic sailfish	Agulhão-chato; Agulhão-de-vela	NE		x	x					0,06
Labridae	*Bodianus rufus* (Linnaeus, 1758)	Spanish hogfish	Budião-perua-choca; Budião-papagaio; Papagaio; Bobó-papagaio	LC		x	x					0,28
Labrisomidae	*Labrisomus nuchipinnis* (Quoy & Gaimard, 1824)	Hairy blenny	Macaco	NE								0,00
Lamnidae	*Carcharodon carcharias* (Linnaeus, 1758)	White shark	Cação-espelho; Cação-branco; Tubarão-branco	VU		x	x					0,44
Lamnidae	*Isurus oxyrinchus* (Rafinesque, 1810)	Shortfin mako	Cação-cavala; Tubarão-cavala	VU		x	x					0,33
Lobotidae	*Lobotes surinamensis* (Bloch, 1790)	Tripletail	Peixe-sono; Dorminhoco	NE		x	x					0,06
Lutjanidae	*Lutjanus analis* (Cuvier, 1828)	Mutton snapper	Cioba; Ciquira	VU		x	x					1,69
Lutjanidae	*Lutjanus* spp.	Dog snapper	Baúna; Vermelha; Dentão; Carapitanga	NE		x	x					1,39
Lutjanidae	*Lutjanus synagris* (Linnaeus, 1758)	Lane snapper	Ariacó	NE		x	x					0,78
Lutjanidae	*Lutjanus vivanus* (Cuvier, 1828)	Silk snapper	Pargo-olho-de-vidro	NE		x	x					0,72
Lutjanidae	*Lutjanus buccanella* (Cuvier, 1828)	Blackfin snapper	Pargo-boca-negra	NE		x	x					0,67
Lutjanidae	*Lutjanus griseus* (Linnaeus, 1758)	Grey snapper	Cambuba; Caranha	NE		x	x		x			0,64
Lutjanidae	*Rhomboplites aurorubens* (Cuvier, 1829)	Vermillion snapper	Pargo-piranga; Pargo-pinanga; Pargo-pininga	NE	x	x	x					0,33
Lutjanidae	*Etelis oculatus* (Valenciennes, 1828)	Queen snaper	Mariquitão; Pargo-Mariquitão	NE		x	x					0,28
Lutjanidae	*Lutjanus* spp.		Parguina	Sem avaliação		x	x					0,11
Lutjanidae	*Ocyurus chrysurus* (Bloch, 1791)	Yellowtail snapper	Guaiúba-amarela; Guaiúba-paiguina	NE	x	x	x					0,08
Malacanthidae	*Malacanthus plumieri* (Bloch, 1786)	Sand tilefish	Pirá	NE		x	x					0,44
Megalopidae	*Megalops atlanticus* (Valenciennes, 1847)	Tarpon	Camurupim	VU		x	x	x	x			0,28
Monacanthidae	*Aluterus* spp.	Dotterel filefish	Cangulo-fóia; Cangulo-folha; Cangulo-seda	NE		x	x					0,44
Monacanthidae	*Monacanthus ciliatus* (Mitchill, 1818)	Fringed filefish	Cangulo-de-areia; Cangulo-peruá	NE		x	x	x				0,19
Mugilidae	*Mugil* spp.		Zereda; Olho-preto; Saúna;Tamatarana; Tainha; Curimã; Saúna-seleste; Tainha-olho-branco; Saúna-olho-branco; Tainha-olho-de-fogo; Tainha-olho-negro; Tainha-parati	Sem avaliação		x	x					1,22
Mullidae	*Pseudupeneus maculatus* (Bloch, 1793)	Spotted goadtifsh	Saramonete	NE		x	x					0,67
Mullidae	*Mulloidichthys martinicus* (Cuvier, 1829)	Yellow goatfish	Saramonete-rei	NE		x	x					0,06
Muraenidae	*Gymnothorax funebris* (Ranzani, 1839)	Green moray	Moréia-verde	NE		x	x					0,33
Muraenidae	*Gymnothorax moringa* (Cuvier, 1829)	Spotted moray	Moréia-pintada	NE		x	x					0,33
Muraenidae	*Gymnothorax ocellatus* (Agassiz, 1831)	Caribbean ocellated moray	Moréia-pintada	NE		x	x					0,33
Muraenidae	*Gymnothorax* spp.	Goldentail moray	Moréia-preta	NE		x	x					0,17
Myliobatidae	*Aetobatus narinari* (Euphrasen, 1790)	Spotted eagle ray	Arraia-pintada; Arraia-malhada; Arraia-pinta-de-manga; Arraia-chita	NT		x	x	x				1,17
Myliobatidae	*Manta birostris* (Walbaum, 1792)	Giant manta	Arraia-dois-chifres; Arraia-jamanta; Arraia-morcego	VU		x	x					0,56
Myliobatidae	*Rhinoptera bonasus* (Mitchill, 1815)	Cownose ray	Arraia-boca-de-gaveta; arraia-gaveta	NT		x	x					0,28
Narcinidae	*Narcine* spp.	Lesser electric ray	Treme-treme	CR								0,00
Ogcocephalidae	*Ogcocephalus vespertilio* (Linnaeus, 1758)	Seadevil	Cachimbo; Cachimbau	NE								0,00
Ostraciidae	*Lactophrys trigonus* (Linnaeus, 1758)	Buffalo trunkfish	Baiacu-caixão	NE								0,00
Polynemidae	*Polydactylus oligodon* (Günther, 1860)	Littlescale threadfin	Barbudo	NE		x	x					0,33
Polynemidae	*Polydactylus virginicus* (Linnaeus, 1758)	Barbu	Barbudo	NE		x	x					0,33
Pomacanthidae	*Pomacanthus arcuatus* (Linnaeus, 1758)	Gray angelfish	Parum-preto; Peixe-vidro; Quebra-pedra	LC		x	x					0,11
Pomacentridae	*Abudefduf saxatilis* (Linnaeus, 1758)	Sergeant-major	Saberé; Saberé-rajado; Sargentinho	NE		x	x	x			x	0,36
Pomacentridae	*Stegastes pictus* (Castelnau, 1855)	Yellowtip damselfish	Castanheta	NE		x	x					0,11
Pomatomidae	*Pomatomus saltatrix* (Linnaeus, 1766)	Bluefish	Enchova; Anchova	NE	x	x	x					0,33
Priacanthidae	*Priacanthus arenatus* (Cuvier, 1829)	Atlantic bigeye	Cantante	NE		x	x					0,17
Pristigasteridae	*Pellona harroweri* (Fowler, 1917)	American coastal pellona	Sardinha-berimberim	NE		x	x					0,06
Rachycentridae	*Rachycentron canadum* (Linnaeus, 1766)	Cobia	Beijupirá; cação-de-escama	NE		x	x					0,83
Rhicodontidae	*Rhincodon typus* (Smith, 1828)	Whale shark	Tubarão-baleia; Tubarão-cachalote	VU								0,00
Rhinobatidae	*Rhinobatos percellens* (Walbaum, 1792)	Chola guitarfish	Cação-viola; Viola	NT		x						0,11
Scaridae	*Scarus trispinosus* (Valenciennes, 1840)	Greenback parrotfish	Bobó-espinha-verde; Budião-azul; Budião-bico-verde; Budião-verde; Bobó-bico-verde	EN		x	x					0,72
Scaridae	*Scarus taeniopterus* (Lesson, 1829)	Princess parrotfish	Budião	LC		x	x					0,22
Scaridae	*Scarus zelindae* (Moura, Figueiredo & Sazima, 2001)	Zelinda's parrotfish	Budião	DD		x	x					0,22
Scaridae	*Sparisoma axillare* (Steindachner, 1878)	Gray parrotfish	Batata; Batatoa; Boboa; Bobó-batatão; Bobó-cabeça-seca; Budião-batata; Budião; Budião-rabo-de-forquilha	DD		x	x					0,22
Scaridae	*Sparisoma frondosum* (Agassiz, 1831)	Agassiz’s parrotfish	Budião	DD		x	x					0,22
Scaridae	*Sparisoma radians* (Valenciennes, 1840)	Bucktooth parrotfish	Batata; Batatoa; Boboa; Bobó-batatão; Bobó-cabeça-seca; Budião-batata; Budião	LC		x	x					0,22
Scaridae	*Sparisoma amplum* (Ranzani, 1841)	Reef parrotfish	Budião-rabo-de-forquilha	LC		x	x					0,06
Sciaenidae	*Cynoscion leiarchus* (Cuvier, 1830)	Smooth weakfish	Pescada-branca	NE		x	x					0,83
Sciaenidae	*Cynoscion virescens* (Cuvier, 1830)	Green weakfish	Pescada-bacalhau; Pescada-camuçu; comeocu; Pescada-cangussu; Pescada-muçu; Pescada-curuvina; Pescada-cabeça-de-cobra; Pescada-cururuca	NE		x	x					0,83
Sciaenidae	*Cynoscion acoupa* (Lacepède, 1801)	Acoupa weakfish	Pescada-amarela	LC		x	x					0,67
Sciaenidae	*Larimus breviceps* (Cuvier, 1830)	Shorthead drum	Boca-mole	NE		x	x					0,61
Sciaenidae	*Micropogonias furnieri* (Desmarest, 1823)	Whitemouth croaker	Curuca; Cururuca; Corvina	NE	x	x	x					0,61
Sciaenidae	*Paralonchurus brasiliensis* (Steindachner, 1875)	Banded croaker	Coróqui-de-barbela; Pescada-perna-de-moça	NE		x	x					0,33
Sciaenidae	*Micropogonias undulatus* (Linnaeus, 1766)	Atlantic croaker	Pescada-perna-de-moça	NE		x	x					0,17
Sciaenidae	*Isopisthus parvipinnis* (Cuvier, 1830)	Bigtooth corvina	Pescada-chata	NE		x	x					0,11
Sciaenidae	*Stellifer microps* (Steindachner, 1864)	Smalleye stardrum	Pescada-curuvina; Pescada-cabeça-de-cobra; Pescada-cururuca	NE		x	x					0,11
Sciaenidae	*Cynoscion jamaicensis* (Vaillant & Bocourt, 1883)	Jamaica weakfish	Goete	NE								0,00
Sciaenidae	*Cynoscion microlepidotus* (Cuvier, 1830)	Smallscale weakfish	Pescada-de-dente	NE								0,00
Scombridae	*Acanthocybium solandri* (Cuvier, 1832)	Wahoo	Cavala-impim; cavala-impinge; cavala-preta	LC		x	x			x		1,25
Scombridae	*Scomberomorus cavalla* (Cuvier, 1829)	King mackerel	Cavala-branca; cavala-perna-de-moça	LC		x	x					1,22
Scombridae	*Scomberomorus regalis* (Bloch, 1793)	Cero	Serra	LC		x	x					1,06
Scombridae	*Thunnus albacares* (Bonnaterre, 1788)	Yellowfin tuna	Albacora-de-aba-amarela; Albacora-gaia-amarela; Albacora-amarela; Albacora-de-lajo	NT		x	x					0,83
Scombridae	*Thunnus obesus* (Lowe, 1839)	Bigeye tuna	Albacora-de-aba-amarela; Albacora-gaia-amarela; Albacora-amarela	VU		x	x					0,83
Scombridae	*Thunnus atlanticus* (Lesson, 1831)	Blackfin tuna	Albacora-cachorro; Albacora-preta	LC		x	x					0,78
Scombridae	*Scomberomorus brasiliensis* (Collette, Russo & Zavala-Camin, 1978)	Serra Spanish mackerel	Serra-pininga; Serra-pinta-amarela	LC		x	x					0,44
Scombridae	*Auxis rochei* (Risso, 1810)	Bullet tuna	Atum; Bonito	LC		x	x					0,33
Scombridae	*Auxis thazard* (Lacepède, 1800)	Frigate tuna	Atum; Bonito	LC		x	x					0,33
Scombridae	*Euthynnus alletteratus* (Rafinesque, 1810)	Little tunny	Atum; Bonito	LC		x	x					0,33
Scombridae	*Katsuwonus pelamis* (Linnaeus, 1758)	Skipjack tuna	Atum; Bonito	LC		x	x					0,33
Scombridae	*Sarda sarda* (Bloch, 1793)	Atlantic bonito	Atum; Bonito	LC		x	x					0,33
Scombridae	*Scomber colias* (Gmelin, 1789)	Atlantic chub mackerel	Cavalinha; Cavalinha-do-sul	LC		x	x					0,22
Scombridae	*Thunnus alalunga* (Bonnaterre, 1788)	Blackfin tuna	Albacora-branca; Albacora-legítima	NT		x	x					0,06
Scombridae	*Thunnus thynnus* (Linnaeus, 1758)	Atlantic bluefin tuna	Albacora-maguru	EN		x	x					0,06
Scorpaenidae	*Scorpaena brasiliensis* (Cuvier, 1829)	Barbfish	Aniquim	NE		x						0,22
Scorpaenidae	*Scorpaena plumieri* (Bloch, 1789)	Spotted scorpionfish	Aniquim; Beatriz	NE		x						0,22
Scorpaenidae	*Scorpaenodes* spp.	Reef scorpionfish	Aniquim	NE		x						0,22
Serranidae	*Cephalopholis fulva* (Linnaeus, 1758)	Coney	Piraúna-amarela; Piraúna-flor-de-algodão; Piraúna-perua-choca; Piraúna-vermelha; Piraúna-preta	LC		x	x					1,83
Serranidae	*Mycteroperca venenosa* (Linnaeus, 1758)	Yellowfin grouper	Sirigado; Badejo	NT		x	x					1,22
Serranidae	*Mycteroperca tigris* (Valenciennes, 1833)	Tiger grouper	Sirigado; Badejo	LC		x	x					1,22
Serranidae	*Epinephelus adscensionis* (Osbeck, 1771)	Rock hind	Peixe-gato; Mané-velho; Garoupa-pintada	LC		x	x					0,67
Serranidae	*Mycteroperca acutirostris* (Valenciennes, 1828)	Comb grouper	Sirigado-papuã	LC		x	x					0,50
Serranidae	*Epinephelus itajara* (Lichtenstein, 1822)	Atlantic goliath grouper	Mero	CR		x						0,42
Serranidae	*Mycteroperca microlepis* (Goode & Bean, 1879)	Gag grouper	Sirigado-bico-fino; Sirigado-masca-fumo	LC		x	x					0,22
Serranidae	*Epinephelus guttatus* (Linnaeus, 1758)	Red hind	Garoupa-preta	LC		x	x					0,17
Serranidae	*Alphestes afer* (Bloch, 1793)	Muttom hamlet	Sapê	LC		x	x					0,11
Serranidae	*Epinephelus morio* (Valenciennes, 1828)	Red grouper	Garoupa-branca	NT	x	x	x					0,11
Serranidae	*Rypticus saponaceus* (Bloch & Schneider, 1801)	Greater soapfish	Sabão	NE		x	x					0,11
Serranidae	*Mycteroperca interstitialis* (Poey, 1860)	Yellowmouth grouper	Sirigado-boca-de-sino	VU		x	x					0,06
Sparidae	*Archosargus rhomboidalis* (Linnaeus, 1758)	Western Atlantic seabream	Salema-açu; Sargo	NE		x	x					0,22
Sparidae	*Calamus pennatula* (Guichenot, 1868)	Pluma porgy	Pena-açu	NE		x	x					0,11
Sparidae	*Archosargus probatocephalus* (Walbaum, 1792)	Sheepshead	Sargo	NE		x	x					0,06
Sparidae	*Calamus penna* (Valenciennes, 1830)	Sheepshead porgy	Pena-branca	NE		x	x					0,06
Sphyraenidae	*Sphyraena barracuda* (Edwards, 1771)	Great barracuda	Goiva; Gorana; Bicuda; Barracuda	NE		x	x					1,00
Sphyraenidae	*Sphyraena guachancho* (Cuvier, 1829)	Guachanche barracuda	Goiva; Gorana; Bicuda; Barracuda	NE		x	x					1,00
Sphyraenidae	*Sphyraena sphyraena* (Linnaeus, 1758)	European barracuda	Goiva; Gorana; Bicuda; Barracuda	NE		x	x					1,00
Sphyrnidae	*Sphyrna lewini* (Griffith & Smith, 1834)	Scalloped hammerhead	Cação-panã; martelo; Tubarão martelo; tubarão-panã	EN	x	x	x					1,00
Sphyrnidae	*Sphyrna mokarran* (Rüppell, 1837)	Great hammerhead	Cação-panã; martelo; Tubarão martelo; tubarão-panã	EN		x	x					1,00
Sphyrnidae	*Sphyrna zygaena* (Linnaeus, 1758)	Smooth hammerhead	Cação-panã; martelo; Tubarão martelo; tubarão-panã	VU	x	x	x					1,00
Sphyrnidae	*Sphyrna tiburo* (Linnaeus, 1758)	Bonnethead	Cação-panã-chapéu-redondo; Cação-sombreiro; Tubarão-sombreiro	LC		x	x					0,06
Squalidae	*Squalus cubensis* (Howell Rivero, 1936)	Cuban dogfish	Cação-de-espeto	DD		x	x					0,17
Syngnathidae	*Hippocampus reidi* (Ginsburg, 1933)	Longsnout seahorse	Cavalo-marinho	DD				x	x	x	x	0,31
Synodontidae	*Trachinocephalus myops* (Forster, 1801)	Snakefish	Traíra	NE		x						0,06
Tetraodontidae	*Lagocephalus* spp.	Smooth puffer	Baiacu-guarajuba; Baiacu-garajuba; Baiacu-arara	NE		x	x					0,44
Tetraodontidae	*Sphoeroides* spp.	Bandtail puffer	Baiacu-franguinho; Baiacu-pintadinho; Baiacu-pintado	NE		x	x					0,44
Triakidae	*Mustelus* sp.		Cação-namorado; Cação-banguelo	Sem avaliação		x	x					0,28
Triakidae	*Mustelus* sp.		Cação-manteiga	Sem avaliação		x						0,03
Trichiuridae	*Trichiurus lepturus* (Linnaeus, 1758)	Largehead hairtail	Espada-branca	NE		x	x					0,11
Xiphiidae	*Xiphias gladius* (Linnaeus, 1758)	Swordfish	Agulhão-espadarte; Agulhão-Meca; Meca	LC		x	x					0,17

*Font: fishbase.org

Legend: NE – Not Evaluated; DD – Data Deficient; LC – Least Concern; NT – Near Threatened; VU – Vulnerable; EN – Endangered; CR – Critically Endangered.

F – Food; Co – Commercial; Med – Medicinal; H – Handicrafts; S-R – Spiritual-religious; Aq – Aquarium.

**Table 2:** **Fish species recorded through interviews with marine artisanal fishermen of the Extractive Reserve Batoque, Ceará, Brazil**

**Family**	**Scientific name**	Name in English*	**Local name**	**IUCN (2014)**	**IBAMA (2004)**	**F**	**Co**	**Med**	**H**	**S-R**	**Aq**	**Use values**
Acanthuridae	*Acanthurus chirurgus* (Bloch, 1787)	Doctorfish	Lanceta	LC		x	x					1,03
Albulidae	*Albula vulpes* (Linnaeus, 1758)	Bonefish	Ubarana	NT		x	x					0,49
Albulidae	*Albula nemoptera* (Fowler, 1911)	Threadfin bonefish	Jutubarana; Tijubarana; Gitubarana	DD		x	x					0,46
Ariidae	*Genidens genidens* (Cuvier, 1829)	Guri sea catfish	Bagre-ariaçu; Bagre-giriaçu; giruaçu; juruaçu; Bagre-branco; Bagre-canhacoco; Bagre-mole	LC		x	x					1,92
Ariidae	*Aspistor quadriscutis* (Valenciennes, 1840)	Bressou sea catfish	Bagre-amarelo; Bagre-mestre-mané; Bagre-mestre-mané-beiçudo; Bagre-boca-de-boi	NE		x	x					1,64
Ariidae	*Bagre bagre* (Linnaeus, 1766)	Coco sea catfish	Bagre-fita	NE		x	x					1,13
Ariidae	*Cathorops spixii* (Agassiz, 1829)	Madamango sea catfish	Bagre-bandim; Bagre-manguim	NE		x	x					0,62
Ariidae	*Sciades herzbergii* (Bloch, 1794)	Pemecou sea catfish	Bagre-camboeiro; Bagre-cambuim	NE		x	x					0,41
Aulostomidae	*Aulostomus maculatus* (Valenciennes, 1841)	Trumpetfish	Trombeta	NE		x	x					0,13
Balistidae	*Canthidermis sufflamen* (Mitchill, 1815)	Ocean triggerfish	Cangulo-guerra-de-garoupa; Cangulo-rabo-de-garoupa; Cangulo-garoupa; Cangulo-preto	NE		x	x		x			1,28
Balistidae	*Melichthys niger* (Bloch, 1786 )	Black triggerfish	Cangulo-guerra-de-garoupa; Cangulo-rabo-de-garoupa; Cangulo-garoupa; Cangulo-preto	NE		x	x		x			1,28
Balistidae	*Balistes capriscus* (Gmelin, 1788)	Grey triggerfish	Cangulo-fernando; Cangulo-fernandi; Cangulo-branco; Cangulo-papo-branco	NE	x	x	x		x			1,23
Balistidae	*Balistes vetula* (Linnaeus, 1758)	Queen triggerfish	Cangulo-amarelo; Cangulo-verdadeiro; cangulo-do-papo-amarelo; Cangulo-papo-louro; Cangulo-azul	VU		x	x	x	x			1,13
Batrachoididae	*Amphichthys cryptocentrus* (Valenciennes, 1837)	Bocon toadfish	Pacamon; Pocomão	LC		x	x					0,72
Batrachoididae	*Batrachoides surinamensis* (Bloch & Schneider, 1801)	Pacuma toadfish	Pacamon; Pocomão	NE		x	x					0,72
Batrachoididae	*Thalassophryne nattereri* (Steindachner, 1876)	Trinidad Tob	Pacamon; Pocomão	NE		x	x					0,72
Belonidae	*Platybelone argalus* (Lesueur, 1821)	Keeltail needlefish	Zambaia-cachorro	LC		x	x					0,69
Belonidae	*Strongylura marina* (Walbaum, 1792)	Atlantic needlefish	Zambaia-azul; Agulha-torta	LC		x	x					0,64
Belonidae	*Tylosurus crocodilus* (Péron & Lesueur, 1821)	Hound needlefish	Zambaia-roliço	NE		x	x					0,49
Belonidae	*Ablennes hians* (Valenciennes, 1846)	Flat needlefish	Zambaia-do-alto; Zambaia-fino; Zambaia-largo; Zambaia-sardinhado	NE		x						0,26
Belonidae	*Strongylura timucu* (Walbaum, 1792)	Timucu	Zambaia-roliço	NE		x	x					0,49
Bothidae	*Bothus* spp.	Plate fish	Sóia	NE								0,00
Carangidae	*Elagatis bipinnulata* (Quoy & Gaimard, 1825)	Rainbow runner	Arabaiana; Guaxum; Guaxumba	NE		x	x					1,36
Carangidae	*Caranx bartholomaei* (Cuvier, 1833)	Yellow jack	Garajuba-amarela	NE		x	x					1,23
Carangidae	*Caranx lugubris* (Poey, 1860)	Black jack	Ferreiro; Garajuba-preta	NE		x	x					1,10
Carangidae	*Seriola lalandi* (Valenciennes, 1833)	Yellowtail amberjack	Arabaiana-pintada	NE		x	x					1,08
Carangidae	*Caranx ruber* (Bloch, 1793)	Bar jack	Garajuba-branca	NE		x	x					0,97
Carangidae	*Caranx latus* (Agassiz, 1831)	Horse-eye jack	Garacimbora; Aracimbora; Garachimbora; Guachimbora; Xaréu-cavala	NE		x	x					0,77
Carangidae	*Caranx hippos* (Linnaeus, 1766)	Crevalle jack	Xaréu; Xerelete	NE		x	x					0,69
Carangidae	*Alectis ciliaris* (Bloch, 1787)	African pompano	Galo-de-penacho; Galo-do-alto; galo-de-fita	LC		x	x					0,67
Carangidae	*Selene vomer* (Linnaeus, 1758)	Lookdown	Galo-de-penacho; Galo-do-alto; galo-de-fita	NE		x	x					0,67
Carangidae	*Chloroscombrus chrysurus* (Linnaeus, 1766)	Atlantic bumper	Pelombeta; Pilombeta; Palombeta	NE	x	x	x					0,64
Carangidae	*Selene setapinnis* (Mitchill, 1815)	Atlanctic moonfish	Galo-da-costa	NE		x	x					0,54
Carangidae	*Selene brownii* (Cuvier, 1816)	Caribbean moonfish	Galo-da-costa	NE		x	x					0,54
Carangidae	*Trachinotus* spp.	Floripa pompano	Pampo; Carabebeu; Garabebeu	NE		x	x					0,44
Carangidae	*Oligoplites palometa* (Cuvier, 1832)	Maracaibo leatherjacket	Tibiro; Timbiro	NE		x	x					0,23
Carangidae	*Oligoplites saliens* (Bloch, 1793)	Castin leatherjacket	Tibiro; Timbiro	NE		x	x					0,23
Carangidae	*Oligoplites saurus* (Bloch & Schneider, 1801)	Leatherjacket	Tibiro; Timbiro	NE		x	x					0,23
Carangidae	*Decapterus macarellus* (Cuvier, 1833)	Mackerel scad	Garapau; Olhão; Oião	NE		x	x					0,15
Carangidae	*Caranx crysos* (Mitchill, 1815)	Blue runner	Chinchá; Chincharro	LC		x	x					0,10
Carangidae	*Trachinotus* sp.		Pelado; Pataca	Sem avaliação		x	x					0,10
Carangidae	*Seriola rivoliana* (Valenciennes, 1833)	Longfin yellowtail	Pitagol; Pitangola; Garajuba-ferrero	NE		x						0,03
Carcharhinidae	*Carcharhinus falciformis* (Müller & Henle, 1839)	Silky shark	Cação-aba-preta; Cação-sicurí; galha-preta; Tubarão-galha-preta; Tubarão-aba-preta; Cação-flamengo	NT		x	x	x				1,38
Carcharhinidae	*Carcharhinus limbatus* (Müller & Henle, 1839)	Blacktip shark	Cação-aba-preta; Cação-sicurí; galha-preta; Tubarão-galha-preta; Tubarão-aba-preta; Cação-flamengo	NT		x	x	x				1,38
Carcharhinidae	*Galeocerdo cuvier* (Péron & Lesueur, 1822)	Tiger shark	Cação-pintadinho; cação-pintado; jaguara; cação-tigre; tubarão-tigre	NT		x	x	x				0,97
Carcharhinidae	*Rhizoprionodon* spp.		Cação-rabo-seco	VU		x	x	x				0,51
Carcharhinidae	*Rhizoprionodon lalandii* (Valenciennes, 1839)	Brazilian sharpnose shark	Cação-verga-de-ouro	DD		x	x	x				0,44
Carcharhinidae	*Rhizoprionodon porosus* (Richardson, 1836)	Caribeean sharpnose Shark	Cação-verga-de-ouro	LC		x	x	x				0,44
Carcharhinidae	*Carcharhinus obscurus* (LeSueur, 1818)	Dusky shark	Cação fi-d'água; Cação-fidalgo	VU		x	x	x				0,13
Carcharhinidae	*Carcharhinus* spp.		Cação-lombo-preto	Sem avaliação		x	x	x				0,13
Carcharhinidae	*Negaprion brevirostris* (Poey, 1868)	Lemon shark	Tubarão-papa-terra	NT		x		x				0,08
Centropomidae	*Centropomus ensiferus* (Poey, 1860)	Swordspine snook	Camurim-branco	NE		x	x					0,85
Centropomidae	*Centropomus pectinatus* (Poey, 1860)	Tarpon snook	Camurim-suvela; Camurim-gaia	NE		x	x					0,77
Centropomidae	*Centropomus parallelus* (Poey, 1860)	Fat snook	Camurim-amarelo	NE		x	x					0,64
Centropomidae	*Centropomus undecimalis* (Bloch, 1792)	Common snook	Camurim-preto	NE		x						0,03
Chaetodontidae	*Chaetodon* spp.	Spotfin butterflyfish	Parum-jandáia; Peixe-prato; Pintado	LC		x	x					0,33
Clupeidae	*Harengula jaguana* (Poey, 1865)	Scaled herring	Sardinha-cascuda; Sardinha-casca-grossa	NE		x	x	x				1,46
Clupeidae	*Opisthonema oglinum* (Lesueur, 1818)	Atlantic thread herring	Sardinha-azul	NE		x	x	x				0,08
Clupeidae	*Sardinella brasiliensis* (Steindachner, 1879)	Brazilian sardinella	Sardinha-roliça	NE		x	x					0,05
Coryphaenidae	*Coryphaena equiselis* (Linnaeus, 1758)	Pompano dolphinfish	Dourado	LC		x	x					1,33
Coryphaenidae	*Coryphaena hippurus* (Linnaeus, 1758)	Common dolphinfish	Dourado	LC		x	x					1,33
Cynoglossidae	*Symphurus* spp.	Spottedfin tonguefish	Sóia-linguado; Linguado	NE								0,00
Dactylopteridae	*Dactylopterus volitans* (Linnaeus, 1758)	Flying gurnard	Avuador-carga-de-palha	NE		x	x					0,05
Dasyatidae	*Dasyatis americana* (Hildebrand & Schroeder, 1928)	Southern stingray	Arraia-bico-de-remo	DD		x	x					0,77
Dasyatidae	*Dasyatis guttata* (Bloch & Schneider, 1801)	Longnose stingray	Arraia-couro-de-lixa; Arraia-verdadeira; Arraia-couro-verde	DD		x	x					0,59
Dasyatidae	*Dasyatis* sp.		Arraia-de-pedra	LC		x	x					0,31
Dasyatidae	*Dasyatis* sp.		Arraia-verdadeira; Arraia-couro-verde	Sem avaliação		x	x					0,26
Dasyatidae	*Dasyatis marianae* (Gomes, Rosa & Gadig, 2000)	Brazilian large-eyed stingray	Arraia-do-oião; Arraia-oiuda	DD		x	x					0,26
Diodontidae	*Diodon hystrix* (Linnaeus, 1758)	Spot-fin porcupinefish	Baiacu-graviola; Baiacu-espinho	NE		x	x					0,49
Diodontidae	*Chilomycterus antillarum* (Jordan & Rutter, 1897)	Web burrfish	Baiacu-espinho; Baiacu-bola	NE		x	x					0,15
Diodontidae	*Chilomycterus spinosus* (Linnaeus, 1758)		Baiacu-espinho	NE		x	x					0,15
Echeneidae	*Echeneis naucrates* (Linnaeus, 1758)	Live sharksucker	Piolho	NE		x	x	x				1,00
Echeneidae	*Remora remora* (Linnaeus, 1758)	Shark sucker	Piolho	NE		x	x	x				1,00
Echinorhinidae	*Echinorhinus brucus* (Bonnaterre, 1788)	Bramble shark	Peixe-prego	DD		x	x					0,18
Engraulidae	*Lycengraulis grossidens* (Spix & Agassiz, 1829)	Atlantic sabretooth anchovy	Arem	NE		x	x					0,10
Engraulidae	*Lycengraulis batesii* (Günther, 1868)	Bates' sabretooth anchovy	Arem	NE		x	x					0,10
Engraulidae	*Anchoa januaria* (Steindachner, 1879)	Rio anchovy	Manjuba	NE		x						0,05
Engraulidae	*Anchoa tricolor* (Spix & Agassiz, 1829)	Piquitinga anchovy	Manjuba	NE		x						0,05
Ephippidae	*Chaetodipterus faber* (Broussonet, 1782)	Atlantic spadefish	Enxada; Parum-branco	NE		x	x					0,33
Exocoetidae	*Hirundichthys rondeletii* (Valenciennes, 1847)	Black wing flyingfish	Avuador-tainha	LC		x	x					0,36
Exocoetidae	*Exocoetus volitans* (Linnaeus, 1758)	Tropical two-wing flyingfish	Avuador-do-alto; Peixe-avuador-grande	NE		x	x					0,10
Exocoetidae	*Hirundichthys affinis* (Günther, 1866)	Fourwing flyingfish	Avuador-da-pesca; Peixe-avuador-pequeno	NE		x	x					0,05
Gempylidae	*Gempylus serpens* (Cuvier, 1829)	Snake mackerel	Espada; Peixe-espada	NE		x	x					0,41
Gerreidae	*Diapterus auratus* (Ranzani, 1842)	Irish mojarra	Caratinga; Carapeba	NE		x	x					0,31
Gerreidae	*Diapterus rhombeus* (Cuvier, 1829)	Caitipa mojarra	Carapeba	NE		x	x					0,26
Gerreidae	*Eucinostomus* sp.	Slender mojarra	Carapicu	NE		x	x					0,10
Gerreidae	*Eucinostomus havana* (Nichols, 1912)	Bigeye mojarra	Carapicu-roliço	NE		x	x					0,05
Gerreidae	*Eucinostomus gula* (Quoy & Gaimard, 1824)	Jenny mojarra	Carapicu-açu	NE		x	x					0,05
Gerreidae	*Eugerres brasilianus* (Cuvier, 1830)	Brazilian mojarra	Carapeba	NE		x	x					0,05
Gerreidae	*Gerres cinereus* (Walbaum, 1792)	Yellon fin mojarra	Carapicu	NE								0,00
Ginglymostomatidae	*Ginglymostoma cirratum* (Bonnaterre, 1788)	Nurse shark	Cação-lixa	DD		x	x	x	x			0,92
Gymnuridae	*Gymnura micrura* (Bloch & Schneider, 1801)	Smooth butterfly ray	Arraia-manteiga	DD		x	x					0,92
Haemulidae	*Haemulon plumierii* (Lacepède, 1801)	White grunt	Biquara	NE		x	x		x			1,77
Haemulidae	*Haemulon chrysargyreum* (Günther, 1859)	Smallmouth grunt	Sapuruna	NE		x	x					1,41
Haemulidae	*Anisotremus surinamensis* (Bloch, 1791)	Black margate	Salema; Pirambu	NE		x	x					1,23
Haemulidae	*Genyatremus luteus* (Bloch, 1790)	Torroto grunt	Golosa	NE		x	x					1,05
Haemulidae	*Haemulon aurolineatum* (Cuvier, 1830)	Tomtate grunt	Xira	NE		x	x					1,03
Haemulidae	*Pomadasys corvinaeformis* (Steindachner, 1868)	Roughneck grunt	Coró-branco	NE		x	x					0,97
Haemulidae	*Conodon nobilis* (Linnaeus, 1758)	Barred grunt	Coró-amarelo; Coró-rajado; Coró-marinheiro; Coróqui-amarelo	NE		x	x					0,87
Haemulidae	*Haemulon steindachneri* (Jordan e Gilbert, 1882)	Chere-chere grunt	Macasso; Omacasso	LC		x	x					0,79
Haemulidae	*Orthopristis ruber* (Cuvier, 1830)	Corocoro grunt	Cabeça-de-coco; cabeça-dura; Canguito	NE		x	x					0,64
Haemulidae	*Haemulon squamipinna* (Rocha & Rosa, 1999)		Sapuruna-preta; Xila grande; Xira-amarela	NE		x	x					0,59
Haemulidae	*Haemulon parra* (Desmarest, 1823)	Sailor's grunt	Cambuba	NE		x	x					0,49
Haemulidae	*Anisotremus virginicus* (Linnaeus, 1758)	Porkfish	Frade	NE		x	x					0,31
Haemulidae	*Haemulon album* (Cuvier, 1830)	White margate	Sapuruna-branca	NE		x	x					0,13
Haemulidae	*Haemulon macrostomum* (Günther, 1859)	Spanish grunt	Cavalo-pedrez	NE		x	x					0,05
Hemiramphidae	*Hemiramphus balao* (Lesueur, 1821)	Balao halfbeak	Agulha-azul; Agulha-preta	NE		x	x					0,62
Hemiramphidae	*Hyporhamphus roberti* (Valenciennes, 1847)	Slender halfbeak	Agulha-helena; Agulha-branca	LC		x	x					0,62
Holocentridae	*Holocentrus adscensionis* (Osbeck, 1765)	Squilrrelfish	Mariquita; jaguriçá; Mariquita-verdadeira	NE		x	x					1,59
Holocentridae	*Myripristis jacobus* (Cuvier, 1829)	Blackbar soldierfish	Mariquita-china; Piranema	NE		x	x					0,05
Istiophoridae	*Istiophorus albicans* (Latreille, 1804)	Atlantic sailfish	Agulhão-de-vela	NE		x	x					1,03
Labridae	*Bodianus rufus* (Linnaeus, 1758)	Spanish hogfish	Budião-perua-choca; Budião-papagaio; Papagaio; Bobó-papagaio	LC		x	x					0,21
Lamnidae	*Carcharodon carcharias* (Linnaeus, 1758)	White shark	Cação-espelho; Cação-branco; Tubarão-branco	VU		x	x	x				0,74
Lamnidae	*Isurus oxyrinchus* (Rafinesque, 1810)	Shortfin mako	Cação-cavala; Tubarão-cavala	VU		x	x	x				0,18
Lobotidae	*Lobotes surinamensis* (Bloch, 1790)	Tripletail	Chacaruna; Chacarona	NE		x	x					0,23
Lutjanidae	*Lutjanus analis* (Cuvier, 1828)	Mutton snapper	Cioba	VU		x	x					1,74
Lutjanidae	*Ocyurus chrysurus* (Bloch, 1791)	Yellowtail snapper	Guaiúba; Guaiúba-ariacó; Guaiúba-rabo-de-forquilha	NE		x	x					1,64
Lutjanidae	*Lutjanus purpureus* (Poey, 1866)	Southern red snapper	Pargo-verdadeiro	NE	x	x	x					1,26
Lutjanidae	*Lutjanus synagris* (Linnaeus, 1758)	Lane snapper	Ariacó	NE		x	x					1,18
Lutjanidae	*Lutjanus vivanus* (Cuvier, 1828)	Silk snapper	Pargo-vidrado; Pargo-olho-de-vidro	NE		x	x					1,13
Lutjanidae	*Lutjanus* spp.	Dog snapper	Baúna; Vermelha; Dentão; Carapitanga	NE		x	x					0,23
Lutjanidae	*Lutjanus griseus* (Linnaeus, 1758)	Grey snapper	Cambuba; Caranha	NE		x	x					0,18
Lutjanidae	*Etelis oculatus* (Valenciennes, 1828)	Queen snaper	Mariquitão; Pargo-pincel	NE		x	x					0,10
Lutjanidae	*Rhomboplites aurorubens* (Cuvier, 1829)	Vermillion snapper	Pargo-piranga; Pargo-pinanga; Pargo-pininga	NE	x	x	x					0,08
Malacanthidae	*Malacanthus plumieri* (Bloch, 1786)	Sand tilefish	Pirá	NE		x	x					1,18
Megalopidae	*Megalops atlanticus* (Valenciennes, 1847)	Tarpon	Camurupim; Camurupim-china; Pema	VU		x	x	x	x			1,87
Monacanthidae	*Stephanolepis hispidus* (Linnaeus, 1766)	Planehead filefish	Cangulo-velho	NE		x	x		x			0,95
Monacanthidae	*Cantherhines* spp.	American whitespotted filefish	Cangulo-mirim; Cangulo-bicudo; cangulo-pavão	NE		x	x		x			0,64
Monacanthidae	*Monacanthus ciliatus* (Mitchill, 1818)	Fringed filefish	Cangulo-de-areia; Cangulo-peruá	NE		x	x	x	x			0,41
Monacanthidae	*Aluterus* spp.	Dotterel filefish	Cangulo-velho	NE					x			0,03
Mugilidae	*Mugil* spp.		Zereda;Olho-preto;Saúna;Tamatarana; Tainha; Saúna-olho-preto	Sem avaliação		x	x					0,90
Mullidae	*Pseudupeneus maculatus* (Bloch, 1793)	Spotted goadtifsh	Bode; Bode-do-mar	NE		x	x					0,18
Muraenidae	*Gymnothorax moringa* (Cuvier, 1829)	Spotted moray	Moréia-pintada	NE		x	x					1,18
Muraenidae	*Gymnothorax ocellatus* (Agassiz, 1831)	Caribbean ocellated moray	Moréia-pintada	NE		x	x					1,18
Muraenidae	*Gymnothorax* spp.	Goldentail moray	Moréia-preta; moréia-roxa	NE		x	x					1,18
Muraenidae	*Gymnothorax funebris* (Ranzani, 1839)	Green moray	Moréia-verde	NE		x	x					0,87
Myliobatidae	*Aetobatus narinari* (Euphrasen, 1790)	Spotted eagle ray	Arraia-pintada; Arraia-malhada; Arraia-capote; Arraia-chita-de-viúva; Arraia-bico-de-viúva; Arraia-fita-de-viúva	NT		x	x					1,38
Myliobatidae	*Rhinoptera bonasus* (Mitchill, 1815)	Cownose ray	Arraia-boca-de-gaveta; arraia-gaveta	NT		x	x					0,77
Myliobatidae	*Manta birostris* (Walbaum, 1792)	Giant manta	Arraia-jamanta; Arraia-morcego	VU		x	x					0,72
Narcinidae	*Narcine* spp.	Lesser electric ray	Puraquê	CR		x	x	x				0,18
Ophichthidae	*Ophichthus gomesii* (Castelnau, 1855)	Shrimp eel	Muriongo	NE		x	x					0,28
Ophichthidae	*Myrichthys ocellatus* (Lesueur, 1825)	Goldspotted eel	Mututuca	NE		x	x					0,05
Ostraciidae	*Acanthostracion* spp.	Honeycomb cowfish	Baiacu-de-chifre; Baiacu-boim	NE		x	x					0,62
Ostraciidae	*Lactophrys trigonus* (Linnaeus, 1758)	Buffalo trunkfish	Baiacu-caixão; Boim; Baiacu-pardalzinho	NE		x	x		x			0,28
Polynemidae	*Polydactylus oligodon* (Günther, 1860)	Littlescale threadfin	Barbudo	NE		x	x					0,95
Polynemidae	*Polydactylus virginicus* (Linnaeus, 1758)	Barbu	Barbudo	NE		x	x					0,95
Pomacanthidae	*Pomacanthus paru* (Bloch, 1787)	French angelfish	Jandáia; Mocinha; Cará-manissoba; Parum-dourado	LC		x	x					0,54
Pomacanthidae	*Pomacanthus arcuatus* (Linnaeus, 1758)	Gray angelfish	Parum-preto; Peixe-vidro; Jandáia; Quebra-pedra	LC		x	x					0,31
Pomacentridae	*Abudefduf saxatilis* (Linnaeus, 1758)	Sergeant-major	Zefinha	NE		x					x	0,05
Pomacentridae	*Stegastes pictus* (Castelnau, 1855)	Yellowtip damselfish	Patriota	NE		x	x					0,05
Pomatomidae	*Pomatomus saltatrix* (Linnaeus, 1766)	Bluefish	Enchova; Anchova	NE	x	x	x					0,23
Priacanthidae	*Priacanthus arenatus* (Cuvier, 1829)	Atlantic bigeye	Olho-de-boi; Oião; Olhão	NE		x	x					1,18
Pristidae	*Pristis* spp.	Smalltooth sawfish	Cação-espadarte	CR								0,00
Pristigasteridae	*Pellona harroweri* (Fowler, 1917)	American coastal pellona	Sardinha-da-noite	NE		x	x					1,79
Rachycentridae	*Rachycentron canadum* (Linnaeus, 1766)	Cobia	Beijupirá; cação-de-escama	NE		x	x					1,38
Rhicodontidae	*Rhincodon typus* (Smith, 1828)	Whale shark	Tubarão-baleia; Tubarão-cachalote	VU								0,00
Rhinobatidae	*Rhinobatos percellens* (Walbaum, 1792)	Chola guitarfish	Cação-viola; Viola	NT		x	x	x				0,69
Scaridae	*Sparisoma axillare* (Steindachner, 1878)	Gray parrotfish	Batata; Boboa; Budião	DD		x	x					0,62
Scaridae	*Sparisoma radians* (Valenciennes, 1840)	Bucktooth parrotfish	Batata; Boboa; Budião	LC		x	x					0,62
Scaridae	*Scarus taeniopterus* (Lesson, 1829)	Princess parrotfish	Budião	LC		x	x					0,15
Scaridae	*Scarus zelindae* (Moura, Figueiredo & Sazima, 2001)	Zelinda's parrotfish	Budião	DD		x	x					0,15
Scaridae	*Sparisoma frondosum* (Agassiz, 1831)	Agassiz’s parrotfish	Budião	DD		x	x					0,15
Scaridae	*Scarus trispinosus* (Valenciennes, 1840)	Greenback parrotfish	Budião-verde; Bobó-bico-verde	EN		x	x					0,05
Sciaenidae	*Cynoscion leiarchus* (Cuvier, 1830)	Smooth weakfish	Pescada-branca	NE		x	x					1,18
Sciaenidae	*Cynoscion acoupa* (Lacepède, 1801)	Acoupa weakfish	Pescada-cutipa; Pescada-ticupa; Pescada-amarela	LC		x	x					1,10
Sciaenidae	*Paralonchurus brasiliensis* (Steindachner, 1875)	Banded croaker	Judeu	NE		x	x					0,72
Sciaenidae	*Cynoscion virescens* (Cuvier, 1830)	Green weakfish	Pescada-curuvina; Pescada-cabeça-de-cobra; Pescada-cururuca	NE		x	x					0,62
Sciaenidae	*Larimus breviceps* (Cuvier, 1830)	Shorthead drum	Boca-mole	NE		x	x					0,36
Sciaenidae	*Micropogonias furnieri* (Desmarest, 1823)	Whitemouth croaker	Curuca; Cururuca; Corvina	NE	x	x	x					0,36
Sciaenidae	*Cynoscion microlepidotus* (Cuvier, 1830)	Smallscale weakfish	Pescada-de-dente	NE		x	x					0,33
Sciaenidae	*Stellifer rastrifer* (Jordan, 1889)	Rake stardrum	Pescada-cascuda	NE		x	x					0,10
Sciaenidae	*Stellifer microps* (Steindachner, 1864)	Smalleye stardrum	Pescada-cascuda; Pescada-curuvina; Pescada-cabeça-de-cobra; Pescada-cururuca	NE		x	x					0,05
Scombridae	*Scomberomorus regalis* (Bloch, 1793)	Cero	Serra	LC		x	x					1,74
Scombridae	*Auxis rochei* (Risso, 1810)	Bullet tuna	Atum; Bonito	LC		x	x					1,54
Scombridae	*Auxis thazard* (Lacepède, 1800)	Frigate tuna	Atum; Bonito	LC		x	x					1,54
Scombridae	*Euthynnus alletteratus* (Rafinesque, 1810)	Little tunny	Atum; Bonito	LC		x	x					1,54
Scombridae	*Katsuwonus pelamis* (Linnaeus, 1758)	Skipjack tuna	Atum; Bonito	LC		x	x					1,54
Scombridae	*Sarda sarda* (Bloch, 1793)	Atlantic bonito	Atum; Bonito	LC		x	x					1,54
Scombridae	*Thunnus albacares* (Bonnaterre, 1788)	Yellowfin tuna	Albacora-de-lajo	NT		x	x					1,33
Scombridae	*Acanthocybium solandri* (Cuvier, 1832)	Wahoo	Cavala-impim; cavala-impinge; cavala-preta	LC		x	x					1,18
Scombridae	*Scomberomorus cavalla* (Cuvier, 1829)	King mackerel	Cavala-branca; cavala-perna-de-moça	LC		x	x					0,87
Scombridae	*Scomber colias* (Gmelin, 1789)	Atlantic chub mackerel	Cavalinha; Cavalinha-do-sul	LC		x	x					0,15
Scorpaenidae	*Scorpaena brasiliensis* (Cuvier, 1829)	Barbfish	Aniquim	NE		x	x					0,21
Scorpaenidae	*Scorpaena plumieri* (Bloch, 1789)	Spotted scorpionfish	Aniquim	NE		x	x					0,21
Scorpaenidae	*Scorpaenodes* spp.	Reef scorpionfish	Aniquim	NE		x	x					0,21
Serranidae	*Mycteroperca venenosa* (Linnaeus, 1758)	Yellowfin grouper	Sirigado	NT		x	x					1,69
Serranidae	*Mycteroperca tigris* (Valenciennes, 1833)	Tiger grouper	Sirigado	LC		x	x					1,69
Serranidae	*Epinephelus guttatus* (Linnaeus, 1758)	Red hind	Garoupa-preta	LC		x	x					1,18
Serranidae	*Epinephelus morio* (Valenciennes, 1828)	Red grouper	Garoupa-branca	NT	x	x	x					1,18
Serranidae	*Cephalopholis fulva* (Linnaeus, 1758)	Coney	Piraúna-amarela; Piraúna-flor-de-algodão; Piraúna-perua-choca; Piraúna-vermelha	LC		x	x					1,05
Serranidae	*Mycteroperca microlepis* (Goode & Bean, 1879)	Gag grouper	Sirigado-bico-fino	LC		x						0,85
Serranidae	*Mycteroperca bonaci* (Poey, 1860)	Black grouper	Sirigado-preto	NT		x						0,85
Serranidae	*Epinephelus itajara* (Lichtenstein, 1822)	Atlantic goliath grouper	Mero	CR		x	x					0,38
Serranidae	*Rypticus saponaceus* (Bloch & Schneider, 1801)	Greater soapfish	Sabão	NE		x	x					0,28
Serranidae	*Diplectrum formosum* (Linnaeus, 1766)	Sand perch	Jacundá; Jajá	NE		x	x					0,18
Serranidae	*Epinephelus adscensionis* (Osbeck, 1771)	Rock hind	Peixe-gato; Garoupa-pintada	LC		x	x					0,13
Serranidae	*Alphestes afer* (Bloch, 1793)	Muttom hamlet	Sapê	LC		x	x					0,08
Sparidae	*Calamus penna* (Valenciennes, 1830)	Sheepshead porgy	Pena-branca	NE		x	x					0,41
Sparidae	*Calamus pennatula* (Guichenot, 1868)	Pluma porgy	Pena-bode	NE		x	x					0,41
Sparidae	*Archosargus probatocephalus* (Walbaum, 1792)	Sheepshead	Sargo	NE		x	x					0,26
Sparidae	*Archosargus rhomboidalis* (Linnaeus, 1758)	Western Atlantic seabream	Sargo	NE		x	x					0,23
Sphyraenidae	*Sphyraena barracuda* (Edwards, 1771)	Great barracuda	Coroma; Bicuda, Barracuda	NE		x	x					0,56
Sphyraenidae	*Sphyraena guachancho* (Cuvier, 1829)	Guachanche barracuda	Coroma; Bicuda, Barracuda	NE		x	x					0,56
Sphyraenidae	*Sphyraena sphyraena* (Linnaeus, 1758)	European barracuda	Coroma; Bicuda, Barracuda	NE		x	x					0,56
Sphyrnidae	*Sphyrna lewini* (Griffith & Smith, 1834)	Scalloped hammerhead	Cação-panã; martelo; Tubarão martelo; tubarão-panã; Cação-panã-tintureira	EN	x	x	x	x				1,36
Sphyrnidae	*Sphyrna mokarran* (Rüppell, 1837)	Great hammerhead	Cação-panã; martelo; Tubarão martelo; tubarão-panã; Cação-panã-tintureira	EN		x	x	x				1,36
Sphyrnidae	*Sphyrna zygaena* (Linnaeus, 1758)	Smooth hammerhead	Cação-panã; martelo; Tubarão martelo; tubarão-panã; Cação-panã-tintureira	VU	x	x	x	x				1,36
Sphyrnidae	*Sphyrna tiburo* (Linnaeus, 1758)	Bonnethead	Cação-panã-chapéu-redondo; cação-sombreiro; Tubarão-sombreiro	LC		x	x					0,10
Squalidae	*Squalus cubensis* (Howell Rivero, 1936)	Cuban dogfish	Cação-bagre	DD		x	x	x				0,08
Syngnathidae	*Hippocampus reidi* (Ginsburg, 1933)	Longsnout seahorse	Cavalo-marinho	DD				x	x	x	x	0,13
Synodontidae	*Trachinocephalus myops* (Forster, 1801)	Snakefish	Traíra	NE		x	x					0,33
Synodontidae	*Synodus foetens* (Linnaeus, 1766)	Inshore lizardfish	Lagartixa; Lagarto	NE								0,00
Tetraodontidae	*Lagocephalus* spp.	Smooth puffer	Baiacu-guarajuba; Baiacu-garajuba; Baiacu-arara	NE		x	x					0,74
Tetraodontidae	*Sphoeroides* spp.	Bandtail puffer	Baiacu-pintadinho; Baiacu-pintado; Baiacu-da-costa; Baiacu-pardalzinho; Baiacu-listrado	NE		x	x					0,74
Trichiuridae	*Trichiurus lepturus* (Linnaeus, 1758)	Largehead hairtail	Espada; Peixe-espada	NE		x	x					0,41

*Font: fishbase.org

Legend: NE – Not Evaluated; DD – Data Deficient; LC – Least Concern; NT – Near Threatened; VU – Vulnerable; EN – Endangered; CR – Critically Endangered.

F – Food; Co – Commercial; Med – Medicinal; H – Handicrafts; S-R – Spiritual-religious; Aq – Aquarium.

The fishermen cited 13 fish without current use, although some of these had had past use (Tables 1 and 2). One example is the “cação-espadarte” (*Pristis* sp.). According to the reports of the Batoque fishermen, this fish has not been found in the region for more than 40 years, although it used to be caught in large numbers and sold for food and handicraft purposes. Currently, the conservation status of this species is categorized as critical by the IUCN [31].

Citations of uses for food involved 92% of the species recorded in Tamandaré and 96% of species in Batoque. While for commercial purposes, 85% of the recorded species were cited by the Tamandaré fishermen and 92% by the Batoque fishermen. These data reveal that in Batoque, fishermen use a more diverse number of fish for food and selling than in Tamandaré where food consumption and trade are more centered on certain species.

In Tamandaré, fish with more citations for food and commercial use were “arabaiana”, also called locally “gurubatã” or “peixe-rei” (*Elagatis bipinnulata*) (n = 35), “dourado” (*Coryphaena* sp.) (N = 33) and “piraúna” (*Cephalopholis fulva*) (n = 33). In Batoque, the fish with the most citations for food and commercial use were the marine “bagre-giriaçu” (*Genidens genidens*) (n = 38), “sardinha-da-noite” (*Pellona harroweri*) (n = 36), “cioba” (*Lutjanus analis*) (n = 34), “biquara” (*Haemulon plumierii*) (n = 34) and “serra” (*Scomberomorus* sp.) (n = 34).

**Figure 2:**
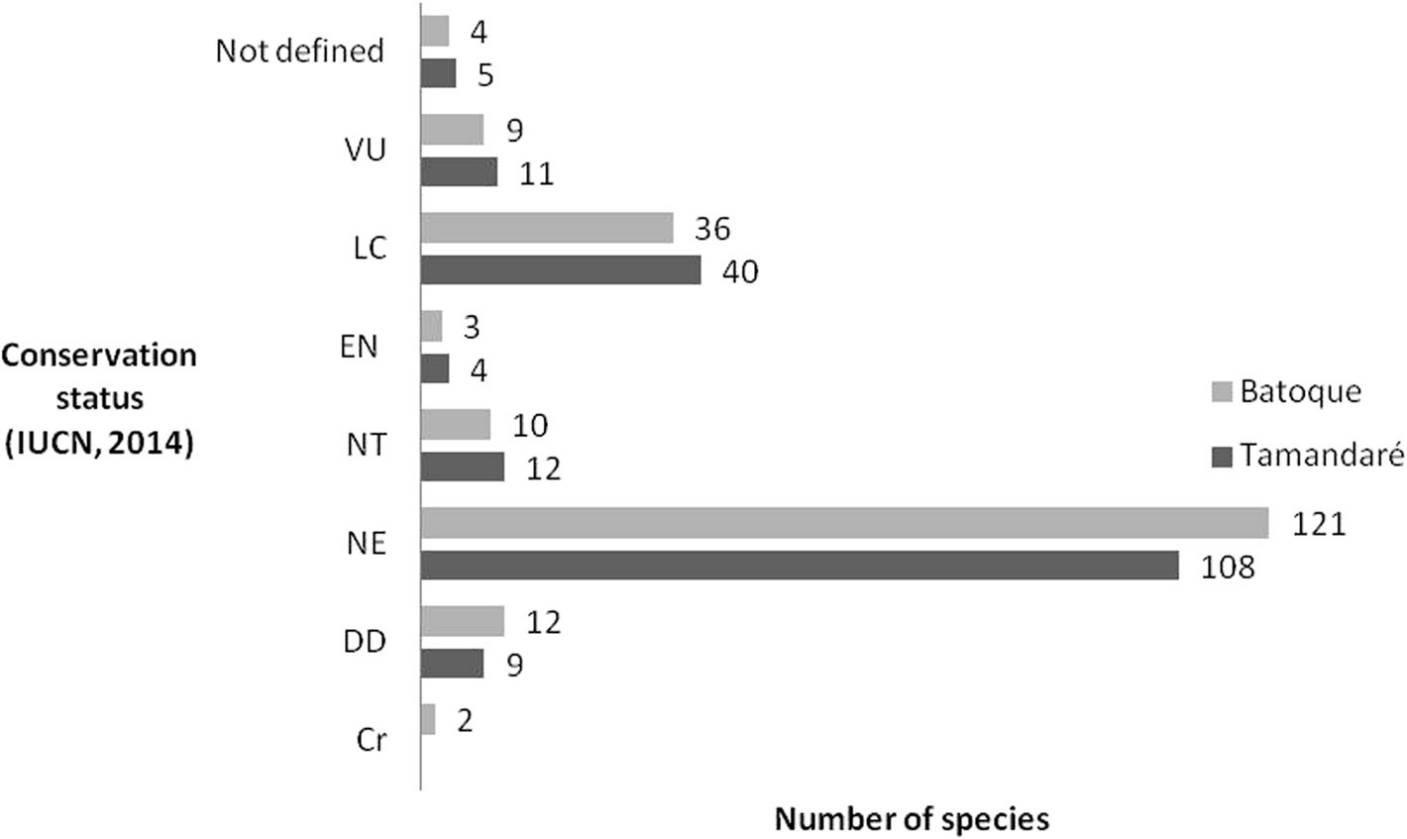
**Conservation status according to IUCN, fish commercialized in Tamandaré (PE) and Batoque (CE).** Legend: NE – Not Evaluated; DD – Data Deficient; LC – Least Concern; NT – Near Threatened; VU – Vulnerable; EN – Endangered; CR – Critically Endangered.

Some of the species recorded for commercial purposes are classified as vulnerable, endangered and critically endangered according to the IUCN Red List [31] (Figure 2). Among the fish sold, the Batoque fishermen cited the “mero” (*Epinephelus itajara*), which has a conservation status of critical [31].

The specie *Lutjanus analis*, known locally as “cioba”, is one of the main commercial fish at Batoque Beach and is classified as vulnerable [31]. The “cação-lixa” (*Ginglymostoma cirratum*), an elasmobranch used for food and sold by the fishermen in both areas is categorized as data deficient by the IUCN [31] and is classified as vulnerable on the IBAMA national red list [32]. It was also found that nine species with commercial use in both areas surveyed (Tables 1 and 2) are present on the national list of species of aquatic invertebrates and overexploited fish or fish threatened by overexploitation [33].

In Tamandaré, some fishermen mentioned that currently the fishing of “mero” (*Epinephelus itajara*) is prohibited, although it was very common more than 10 years. In Batoque, fishermen were unaware that “mero” was a nationally protected fish, as established by IBAMA Ordinance No. 121 of September 20, 2002 [34], regulated by the “Instrução Normativa Interministerial” No. 13, dated October 16, 2012 [35],which prohibits for a period of three years the capture in Brazilian waters of *E. itajara*, popularly known as “mero”, “canapu", “bodete”, “badejão”, “merete” and “merote”.

The fact that the Tamandaré fishermen stated that “mero” fishing was prohibited was explained by the actions of the federal agency Chico Mendes Institute for Conservation of Biodiversity (ICMBio) and Mere Project in Brazil, both based in the city. This project develops conservation policies for the “mero” fish (*E. itajara*) and associated marine environments in several areas on the Brazilian coast, through a network of institutions. At Batoque Beach, ignorance of the law was due to the lack of supervision on site and of any campaign to raise awareness about the ban on fishing of “mero”. It is noteworthy that the capture of this fish, when it occurs at Batoque, is accidental, according to the fishermen.

Regarding fish used for medicinal purposes, six species were recorded in Tamandaré and 26 in Batoque. The fishermen described different ways of preparing fish for medicinal purposes according to the disease being treated (Table 3). Among the fish with the highest number of citations for that purpose, in both communities, was the “baiacu-espinho” (*Chilomycterus antillarum*) and “cavalo-marinho” (*Hippocampus reidi*).

**Table 3:** **Fish used for medicinal purposes by fishermen Beach Tamandaré (PE) and Batoque (CE)**

**Family/Species**	**Local name**	**Number of citations**	**Part used**	**Mode of preparation**	**Illness**
**Balistidae**					
*Balistes vetula* (Linnaeus, 1758)					
**Monacanthidae**					
*Monacanthus ciliatus* (Mitchill, 1818)	*Cangulo*	02	Head	Ingestion	Asthma
*Cantherhines macrocerus* (Hollard, 1853)			Leather		Sexual impotence
**Carcharhinidae**					
*Carcharhinus* sp. (Blainville, 1816)					
*Carcharhinus falciformis* (Müller&Henle, 1839)					
*C. leucas* (Müller&Henle, 1839)					
*C. obscurus* (LeSueur, 1818)					
*C. limbatus* (Müller&Henle, 1839)					
*Galeocerdo cuvier* (Péron&Lesueur, 1822)					
*Rhizoprionodon* spp. (Whitley, 1929)					
*R. porosus* (Richardson, 1836)					
*R. lalandii* (Valenciennes, 1839)					
*Negaprion brevirostris* (Poey, 1868)					
**Ginglymostomatidae**					
*Ginglymostoma cirratum* (Bonnaterre, 1788)	*Cação*	01	Vertebrae	Tea	Osteoporosis
**Lamnidae**					
*Carcharodon carcharias* (Linnaeus, 1758)					
*Isurus oxyrinchus* (Rafinesque, 1810)					
**Rhinobatidae**					
*Rhinobatos percellens (Walbaum, 1792)*					
**Sphyrnidae**					
*Sphyrna lewini* (Griffith & Smith, 1834)					
*S.mokarran* (Rüppell, 1837)					
*S. zygaena* (Linnaeus, 1758)					
**Squalidae**					
*Squalus cubensis* (Howell Rivero, 1936)					
**Megalopidae**					
*Megalops atlanticus* (Valenciennes, 1847)	*Camurupim*	06	Scales	Tea	Asthma
**Clupeidae**					
*Opisthonema oglinum* (Lesueur, 1818)	*Sardinha*	01	Whole body	Ingestion	Osteoporosis
*Harengula jaguana* (Poey, 1865)					
**Diodontidae**					
*Chilomycterus antillarum* (Jordan &Rutter, 1897)	*Baiacu-espinho*	08	Liver	External use	Wound,
*C.spinosus spinosus* (Linnaeus, 1758)			Lard		Lump
**Echeneidae**					
*Echeneis naucrates* (Linnaeus, 1758)	*Piolho*	01	Suction cup (hat)	Tea	Asthma
*Remora remora* (Linnaeus, 1758)					
**Myliobatidae**					
*Aetobatus narinari* (Euphrasen, 1790)	*Arraia-pintada*	06	Tongue	Tea	Asthma
**Narcinidae**					
*Narcine bancrofti* (Griffith & Smith, 1834)	*Puraquê*	01	Lard	External use	Pain,
*N. brasiliensis* (Olfers, 1831)					sore
**Syngnathidae**					
*Hippocampus reidi* (Ginsburg, 1933)	*Cavalo-marinho*	0,12	Whole body	Tea	Asthma

**Figure 3:**
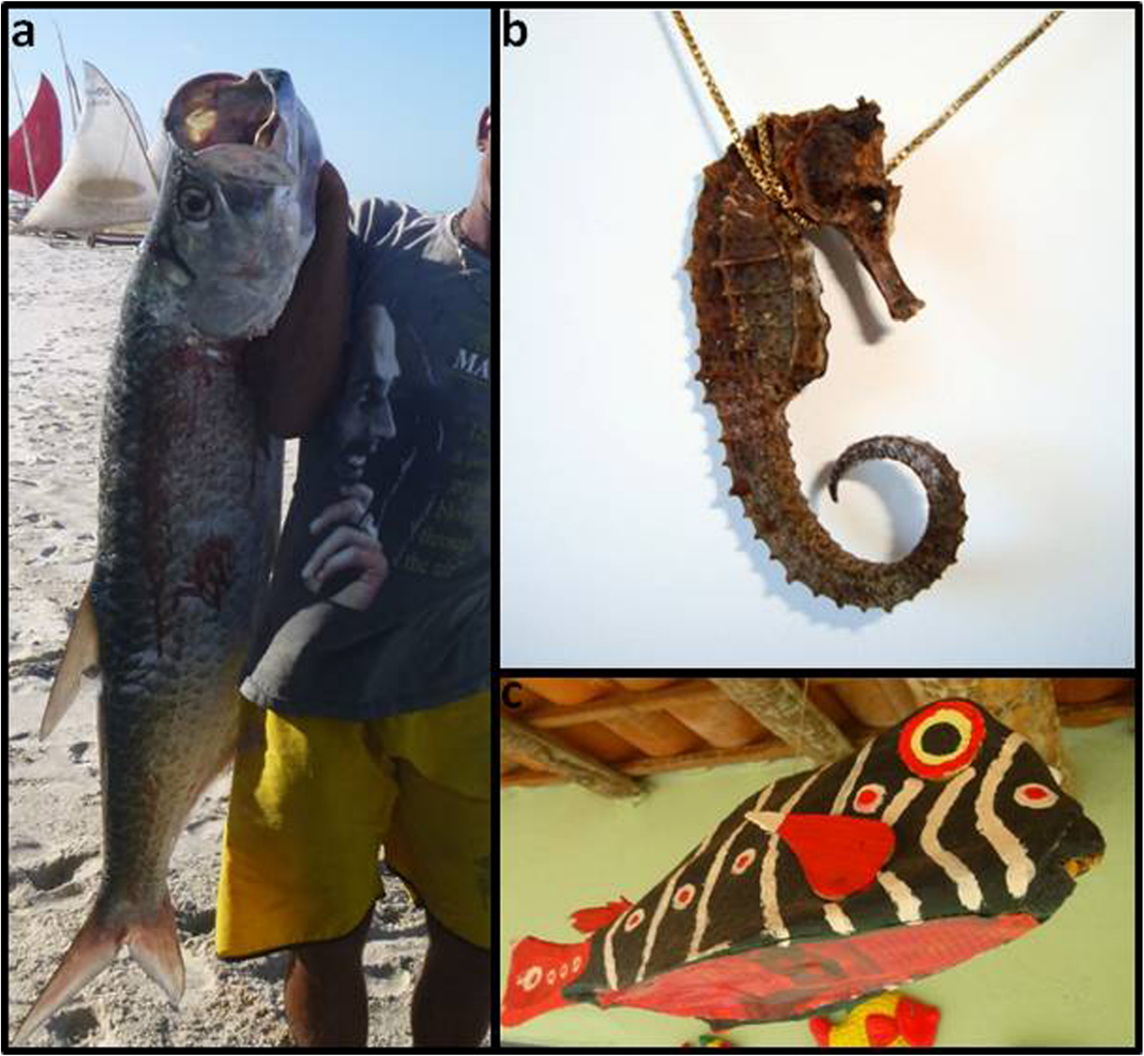
**Fish that provide products with potential use for craft purposes. a)** Fisherman’s Beach Batoque with “camurupim” (*Megalops atlanticus*), whose scales are used to make earrings, curtains and decorative objects. **b)** “Cavalo-marinho” (*Hippocampus reidi*) used with pendant. **c)** “Baiacu-caixão” (*Lactophrys trigonus*) used as a decorative object.

Another mode of use of the fish fauna recorded is related to making crafts (Table 4), for which three species were recorded in Tamandaré and 13 in Batoque, among which the “camurupim” (*Megalops atlanticus*) (Figure 3a) showed a higher number of citations (n = 10). Fishermen acknowledged the use of the scales of this fish to make earrings, curtains and decorative objects, but they claimed that they did not do those themselves. In some cases, the whole fish was used for crafts, such as the “cavalo-marinho” (*H. reidi*), which was killed by asphyxiation, sun-dried and used for decoration, as pendant (Figure 3b) or keychain. The “baiacu-caixão” (*Lactophrys trigonus*), also used whole for making crafts, was killed by asphyxiation and then taxidermied, where the internal organs were removed and the body cleaned with water and internally stuffed with paper or foam. Finally, the fish was sewn and sun-dried, and later, it could be painted and used for decoration (Figure 3c).

**Table 4:** **Fish used for making handicrafts by fishermen Beach Tamandaré (PE) and Batoque (CE)**

**Family/Species**	**Local name**	**Number of citations**	**Part used**
**Balistidae**			
*Balistes vetula* (Linnaeus, 1758).			
*Balistes capriscus* (Gmelin, 1788).			
*Canthidermis sufflamen* (Mitchill, 1815)			
*Melichthys niger* (Bloch, 1786 )			
**Monacanthidae**			
*Monacanthus ciliatus* (Mitchill, 1818)	*Cangulo*	1	Whole body
*Cantherhines* spp. (Swainson, 1839)			
*Aluterus heudelotii* (Hollard, 1855)			
*Aluterus schoepfii* (Walbaum, 1792)			
*Aluterus monoceros* (Linnaeus, 1758)			
*Aluteru sscriptus* (Osbeck, 1765)			
*Stephanolepis hispidus* (Linnaeus, 1766)			
**Ginglymostomatidae**			
*Ginglymostoma cirratum* (Bonnaterre, 1788)	*Cação-lixa*	1	Lard
**Haemulidae**			
*Haemulon plumierii* (Lacepède, 1801)	*Biquara*	1	Whole body
**Lutjanidae**			
*Lutjanus griseus* (Linnaeus, 1758)	*Caranha*	1	Scales
**Megalopidae**			
*Megalops atlanticus* (Valenciennes, 1847)	*Camurupim*	10	Scales
**Ostraciidae**			
*Lactophrys trigonus* (Linnaeus, 1758)	*Baiacu-caixão*	1	Whole body
**Syngnathidae**			
*Hippocampus reidi* (Ginsburg, 1933)	*Cavalo-marinho*	1	Whole body

In addition, the fishermen of the two areas studied mentioned the use of “cavalo-marinho” (*H. reidi*) for magical-religious purposes, where they were sun-dried and used whole as a pendant or kept in the pants pocket. In Tamandaré, one fisherman kept in a small pouch the bony structures from inside the head of the “cavala” (*Acanthocybium solandri*), called “pebbles” (otoliths), which he took while fishing. According to the fishermen, these fish are used as amulets because they bring good luck and good fishing.

The fishermen interviewed acknowledged the use of fish for the aquarium trade, but they did not make that kind of use. The “cavalo-marinho” (*H. reidi*) and species *Abudefduf saxatilis*, called “saberé” by the Tamandaré fishermen and “zefinha” by the Batoque fishermen, were cited as having potential aquarium use.

**Figure 4:**
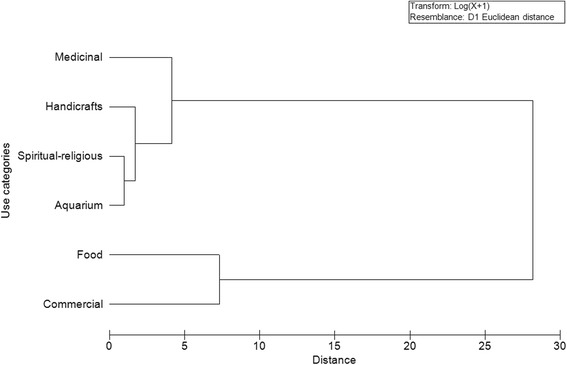
**Dendogram using the Euclidean distance, developed from 207 species listed by fishermen Beach Tamandaré (PE) for each category of use.**

**Figure 5:**
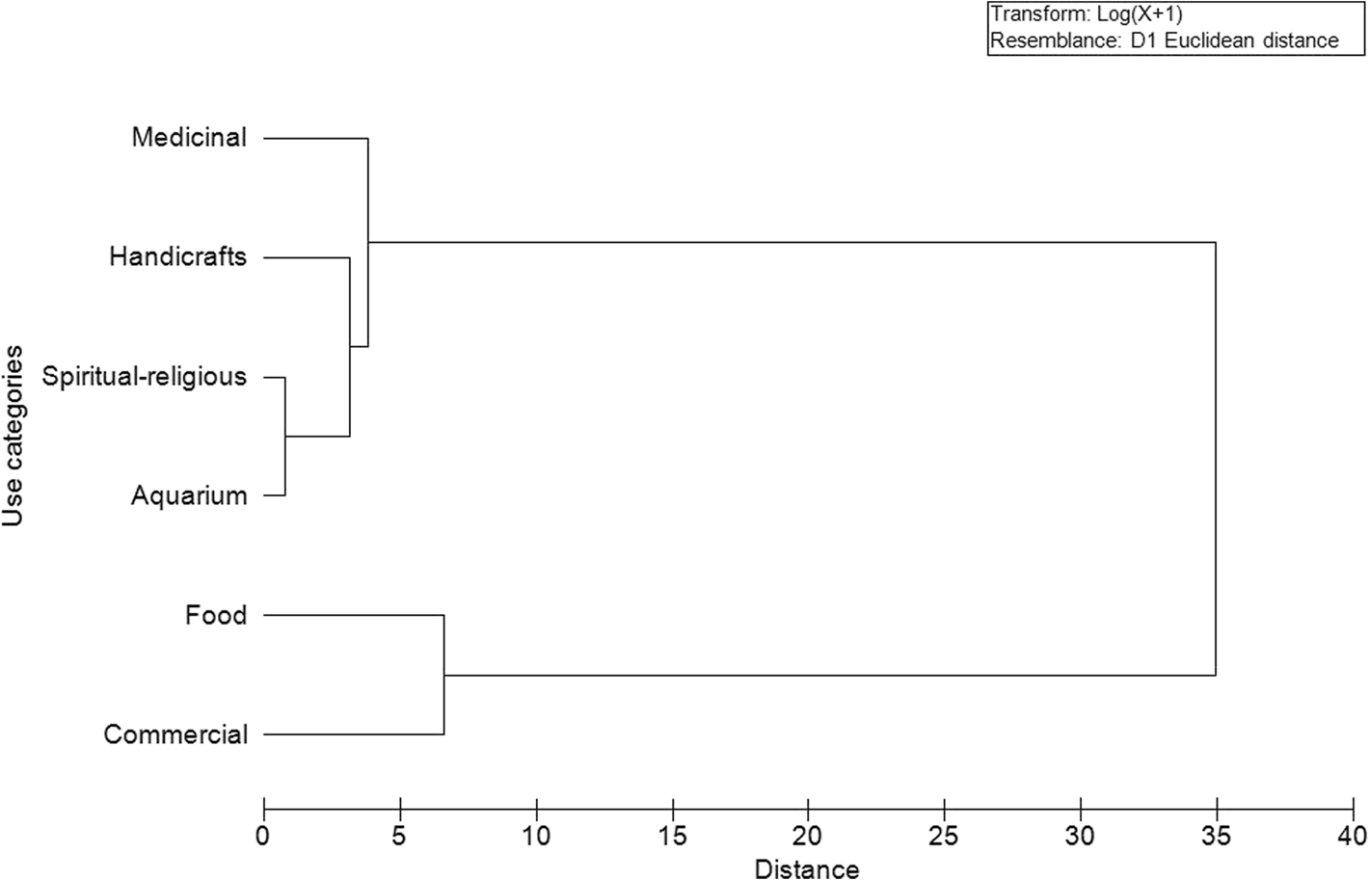
**Dendogram using the Euclidean distance, developed from 209 species listed by fishermen Beach Batoque (CE) for each category of use.**

When evaluating the relationship between the types of use of fish cited by the Tamandaré and Batoque fishermen (Figures 4 and 5), there was a cluster of a greater number of species used for food and trade, to the detriment of species used for other purposes. It was found that this difference in grouping was mainly in the Batoque, where the Euclidean distance was 35 (Figure 5), while in Tamandaré, it was less than 30 (Figure4). This fact is probably due to the greater use of different species in Batoque for food and trade.

In summary, the use of fish cited by fishermen was according to the following standards: i) the fishermen had multiple uses for fish; ii) the primary use was for food; iii) relationships existed between different uses, but the fish used for medicinal purposes, handicrafts, magical-religious purposes and aquariums were not necessarily those used for food and trade.

## Discussion

Socioeconomic data of the fishermen in the areas surveyed were similar to those that have been recorded in other coastal areas of the world and Brazil, where artisanal fishermen are predominantly male, are generally older, and have low levels of education and income [36].

The small number of fishermen under 30 years of age is a trend observed throughout Brazil, where only 22% of the fishermen are under 30 years of age [36]. This may be indicative of young men’s lack of interest [14,37]. On the other hand, many fishermen have been fishing for more than 10 years, highlighting the economic and social importance of this activity, especially in communities with low social indicators, as the case in the study areas. A similar situation was reported elsewhere among the fishermen of Pernambuco State [38] as well as in other fishing communities in Brazil [39-41].

The low level of education among the fishermen interviewed corroborates the numbers from the Registrar General for Fisheries (RGP) of the Ministry of Fisheries and Aquaculture in Brazil [4], which show that 8.1% of registered fishermen are illiterate and that most Brazilian fishermen (75.51%) have only finished elementary school. The results of this research suggest that fishermen who dedicated less of their life to fishing had more opportunities to study, perhaps due to access to schools, which has recently been improved in the areas surveyed. One of the main reasons for dropping out of school may be the need to help support the family, and the lack of incentive to continue studies [42], which directly affects the ability of this working class in social organization.

Although they have low educational level, several studies emphasize that fishermen have ichthyological knowledge [13,14,43]. In this study, we demonstrated the high richness of fish known by the Tamandaré (222 taxa) and Batoque (215 taxa) fishermen, consistent with what has been found in zoological and ethnozoological research conducted in the areas surveyed or in nearby areas. According to the study of marine fish fauna of the Coral Coast Environmental Protected Area, 185 species [44] have been identified. In Ceará, in an ethnotaxonomic study with fishermen of Redonda Beach, at the eastern end of Ceará, 290 species of fish [45] have been identified.

The results of this study indicated that the primary use of the fish fauna recognized by fishermen matches is food, a situation recorded in most ethnoichthyological studies [37,40,46], which are generally aimed at investigating this form of ichthyofauna use. Nevertheless, the products derived from the fish mentioned are also used for other purposes, mainly for commercial food purposes.

Similar to what has been recorded in the fishing communities of North and Northeast Brazil [47], some of the fish fauna of the study areas (30 species) are a source of products used in the preparation of traditional medicines. The number of fish species used in traditional medicine is not surprising, considering their availability and ease of access to freshwater and coastal areas [47,48]. Furthermore, the representativeness of the fish used in traditional medicine has been remarkable, as evidenced by recent reviews on the topic. In Latin America, for example, where at least 584 animals are used for medicinal purposes, 110 are fish species [49]. For this type of use, 93 fish species have been recorded in Brazil [50], of which 58 were recorded in the Northeast region [51].

There was the contrast in the number of medicinal species between the two studies areas, which can be explained by the greater ease of access to conventional medicines in Tamandaré compared to Batoque. This can lead to the replacement of traditional medicine with conventional medicine. However, the common situation in folk medicine was still found to be evident, that is, the overlap between food and medicinal uses. Many fish are consumed for health reasons to prevent or treat illnesses. In a recent review, Alves *et al*. [50] found that animals are used in Brazil as a source of protein and medicine simultaneously and recorded a total of 77 fish species that fit this context.

Products derived from fish populations are also utilized for making handicrafts in the areas surveyed. This practice has been reported in other places in Brazil, where products from marine animals are used for this purpose, in some cases generating income for many people [52,53]. The use of various animal taxa for handicrafts is widespread worldwide practice, which includes about 5,000 species of molluscs, 40 species of coral and unknown numbers of sponges, echinoderms and fish that are part of the global trade in marine souvenirs [54].

In the study areas, the fish fauna also featured magical-religious use. This type of use, although little studied, is widespread in Brazil [55,56]. Magical-religious use involves different animal taxa, as pointed out in recent studies, which revealed that approximately 100 species of animals are used for this purpose in Brazil [50,51,55], including 19 species of fish [50]. Since ancient times, human cultures attributed magical and religious significance to wild and domesticated animals [55-57].

Some products of magical-religious use recorded in the areas surveyed, such as “cavala” (*Acanthocybium solandri*) otoliths, called “pebbles” and used as amulets by fishermen, are similar to those reported elsewhere in the world. According to a study conducted in Baía de Cádiz, Spain, “the bearer of otoliths considers the amulet as a talisman that has properties to ward off evil and curses” [58]. The author also notes that, formerly, the otoliths of the meagre (*Argyrosomus regius*) were carried in cloth bags or loose in pockets as an amulet and that they are currently marketed in the form of rings, earrings and pendants.

The use of fish for the aquarium was also noted by the fishermen, which is not surprising, since the aquarium hobby is enjoyed in many places around the world [59]. In the last two decades, the million-dollar market of ornamental fish showed great expansion [60], and Brazil stands out as one of the five major exporters of tropical fish for aquariums in the world. Although there are no official statistics on the marine ornamental trade, it is estimated that in Brazil, 75 fish species are caught for the aquarium trade, with 26 being endemic [61]. Among the species cited by fishermen in the present study, seahorses were distinguished by their wide use for aquarium purposes, as recorded in other places in Brazil [62]. In addition, *H. reidi* was noted primarily for its multiple use in various locations around the country [39,40,51].

The multiple use of fish in fishing communities is common, as was recorded in the study areas and in various fishing communities [39,40,45-47]. The diversity of uses of ichthyofauna reinforces the importance of fish in the culture, livelihood and economic activities of fishing communities where artisanal fishermen catch fish for different purposes. Understanding these different uses and also the meanings that fish possess within a social context is of utmost importance for the formulation of conservation measures consistent with local realities.

## Implications for conservation

The information obtained from this research can contribute to the preparation of conservation measures directed at endangered species as well as for the creation of marine part of the Extractive Reserve of Batoque and overhaul of the management and administration of fisheries resources of the Coral Coast Environmental Protected Area.

Most fish cited by fishermen for commercial purposes were not evaluated by the International Union for Conservation of Nature and Natural Resources (IUCN), showing a significant gap related to the conservation of fish species that suffer intense fishing pressure. It is recommended to pay special attention to species of the subclass Elasmobranchii (sharks and rays) and families Serranidae (sawfishes and mackerel) and Lutjanidae (snappers), due to the large number of species that are traded and on lists of threatened species. Also, seahorses (*H. reidi*) deserve conservationist attention, because they are listed as data deficient by the IUCN and have been exploited for a variety of uses, which causes strong pressure on the populations of the species.

We emphasize the need for discussion between environmental agencies and fishermen on the conservation status of fish, because conservation measures that aim to ban the fishing of some species, such as the “mero” (*E. itajara*), or the imposition of no fishing in marine areas, has not proven effective, causing conflicts between social and environmental aspects that involve fishing.

The results presented, as well as other ethnoichthyological studies point to the need for greater involvement of fishermen in decisions about the management of fisheries resources, it is increasingly evident that the ecological knowledge of fishermen is critical to the implementation of management plans. Even greater control of illegal fishing and industrial fishing is recommended, since such activities have a known impact on marine fish populations, and have affected artisanal fishing, as pointed out by the fishermen themselves. It is believed that actions considering such recommendations can contribute to the sustainable management of fisheries resources, aimed at the conservation of exploited fishes, as well as the maintenance of coastal artisanal fishing.

As the study sites are inserted in protected areas, it is believed that the actions for the conservation of fishery resources can be more efficient. However, for this to happen, it is necessary a joint action between environmental agencies, governments, researchers and the local community.

## Conclusions

Our results evidence the importance of including artisanal fishermen in pursuit of effectiveness and fishery resources conservation strategies. These workers and their families depend directly on fishing for their social, economic and cultural development. Therefore, the fishing communities have an intrinsic interest in the preservation of the resources they exploit. Many of these communities are included in protected areas and, therefore, fishermen must be involved in the development and implementation of management plans and management of these areas, especially when considering that there are many examples of inefficiency in these management plans and in the conservation of protected areas in Brazil.

The ethnoichthyological studies are useful for understanding the relationship between fishermen and fish as they contain important information for managers of protected areas. Information about the most exploited species, types of uses, overfishing and population decline are essential when searching ways of sustainable management. In areas of this study, for example, we emphasize the need for adjustments in the management of certain species. As for examples, have been the “mero” (*E. itajara*) and the “cavalo-marinho” (*H. reidi*). Beyond these species, ichthyofauna of the groups that deserve conservation attention of management and environmental agencies, sharks and rays are included and also species of Serranidae and Lutjanidae families.

The use and/or the recognition of different fish used by fishermen emphasize the importance of these animals to the culture of fishing communities. Fish are not used by artisanal fishermen and their families only for food consumption and trade, they are also important for medical purposes, for making handicrafts and magic-religious purposes. For this reason, artisanal fishing should not be understood only as a subsistence activity and commercial purposes, but also as a cultural activity. The fish used for aquarium purposes deserve also conservation attention because the aquarium is a commercial practice and that usually involves species that are most vulnerable.
